# Biofertilizer and biocontrol properties of *Stenotrophomonas maltophilia* BCM emphasize its potential application for sustainable agriculture

**DOI:** 10.3389/fpls.2024.1364807

**Published:** 2024-03-04

**Authors:** Pinki Sharma, Rajesh Pandey, Nar Singh Chauhan

**Affiliations:** ^1^ Department of Biochemistry, Maharshi Dayanand University, Haryana, Rohtak, India; ^2^ INtegrative GENomics of HOst-PathogEn (INGEN-HOPE) Laboratory, CSIR-Institute of Genomics and Integrative Biology Council of Scientific and Industrial Research (CSIR-IGIB), Delhi, India; ^3^ Academy of Scientific and Innovative Research (AcSIR), Ghaziabad, India

**Keywords:** biofertilizer, biocontrol agent, wheat rhizosphere, plant growth promotion, genome characterization, sustainable agriculture, comparative genomics

## Abstract

**Introduction:**

Microbial biofertilizers or biocontrol agents are potential sustainable approaches to overcome the limitations of conventional agricultural practice. However, the limited catalog of microbial candidates for diversified crops creates hurdles in successfully implementing sustainable agriculture for increasing global/local populations. The present study aimed to explore the wheat rhizosphere microbiota for microbial strains with a biofertilizer and biocontrol potential.

**Methods:**

Using a microbial culturing-based approach, 12 unique microbial isolates were identified and screened for biofertilizer/biocontrol potential using genomics and physiological experimentations.

**Results and discussion:**

Molecular, physiological, and phylogenetic characterization identified *Stenotrophomonas maltophilia* BCM as a potential microbial candidate for sustainable agriculture. *Stenotrophomonas maltophilia* BCM was identified as a coccus-shaped gram-negative microbe having optimal growth at 37°C in a partially alkaline environment (pH 8.0) with a proliferation time of ~67 minutes. The stress response physiology of *Stenotrophomonas maltophilia* BCM indicates its successful survival in dynamic environmental conditions. It significantly increased (P <0.05) the wheat seed germination percentage in the presence of phytopathogens and saline conditions. Genomic characterization decoded the presence of genes involved in plant growth promotion, nutrient assimilation, and antimicrobial activity. Experimental evidence also correlates with genomic insights to explain the potential of *Stenotrophomonas maltophilia* BCM as a potential biofertilizer and biocontrol agent. With these properties, *Stenotrophomonas maltophilia* BCM could sustainably promote wheat production to ensure food security for the increasing population, especially in native wheat-consuming areas.

## Introduction

Wheat is a principal source of calories and nutritional sustenance for most of the world’s population. The escalating global population poses a formidable challenge to food production systems, necessitating a heightened focus on augmenting wheat production by at least 1.5% per year by 2050 to meet burgeoning dietary demands. We have easily achieved it using fertilizers, hybrid seeds, and pesticides. However, their continuous use has adversely affected soil quality, which limits us from using another chemical-based green revolution. The disruption of soil ecology highlights the urgency for sustainable and integrated interventions to meet requirements without affecting soil ecology. The development of biofertilizers and their integration into agricultural practices pave the way toward achieving growth targets sustainability. Biofertilizer microbial strains are pivotal in enhancing plant growth and productivity through their intricate interactions with the rhizosphere. These strains, often comprising beneficial bacteria or mycorrhizal fungi, contribute to sustainable agriculture by promoting nutrient availability, facilitating nutrient uptake, and inducing plant systemic resistance. Symbiotic microbes like *Rhizobium*, *Sinorhizobium*, *Azoarcus*, *Mesorhizobium*, *Frankia*, *Allorhizobium*, *Bradyrhizobium*, *Burkholderia*, *Azorhizobium*, and *Achromobacter* strains (Nosheen et al., 2021) and free-living microbes like *Azospirillum*, *Azotobacter*, *Azoarcus*, *Gluconacetobacter*, and *Herbaspirillum* (Steenhoudt and Vanderleyden, 2000) have proven their potential to meet plant nitrogen requirements (Shah et al., 2021). The phosphate solubilization potential of *Agrobacterium* sp., *Azotobacter* sp., *Bacillus* sp., *Burkholderia* sp., *Enterobacter* sp., *Erwinia* sp., *Pseudomonas* sp., etc. (Shah et al., 2021) can be employed to meet our plant phosphate requirement. The potassium-solubilizing microbe like *Bacillus edaphicus* (Sheng and He, 2006), *Bacillus megaterium*, *Arthrobacter* sp (Keshavarz Zarjani et al., 2013), and *Paenibacillus glucanolyticus* (Sangeeth et al., 2012) enhances plant production. Various microbes were characterized for their plant growth-promoting potential by plant hormone secretion (Kumar et al., 2023), siderophores generation (Kumar et al., 2023), nutrient assimilation (Fatima and Senthil-Kumar, 2015), and biotic and abiotic stress resistance (Tsukanova et al., 2017). Employment of these microbial biofertilizers could fulfill the plant growth requirements for increased crop production. The wheat yield enhancement also requires employing biocontrol agents to overcome phytopathogen infestation. Wheat phytopathogens *Rhizoctonia solani* and *Fusarium oxysporum* severely affect seed germination and seedling growth (Harris and Moen, 1985; El Chami et al., 2023). It ultimately results in bare patches in crop areas up to 20% (Anees et al., 2010). These pathogens have significantly reduced the production of wheat. Australia’s southern and western cropping regions have documented annual losses of $59 million and $166 million, respectively (Murray and Brennan, 2009). Also, this pathogen has a wide host range (Cook et al., 2002), and thus, it is much more difficult to control. Crop rotation strategy implementation may reduce the fungal infestation to some extent. However, even this strategy reduced grain production (Volsi et al., 2022). Various biocontrol agents like *Trichoderma* sp., Mycorrhizal fungi, and *Pseudomonas fluorescens* demonstrated antagonism toward *Rhizoctonia solani* and *Fusarium oxysporum*. The applicability of these control agents in wheat cultivation lies in their ability to enhance plant defense mechanisms, induce systemic resistance, and compete for resources with pathogenic fungi. Biocontrol agents primarily excel in mitigating phytopathogens; however, their role in directly promoting plant growth in wheat may be comparatively less pronounced.

A limited number of strains are known to exert the dual effect of biocontrol and biofertilization. *Bacillus subtilis* and *Bacillus amyloliquefaciens* represent a group with dual functionality, producing antimicrobial compounds such as polyketides, ribosomal peptides, and bacteriocins for biocontrol and contributing to plant growth promotion through the production of siderophores (Caulier et al., 2019). *Pseudomonas fluorescens* is recognized for biocontrol against various pathogens such as soil-borne *Fusarium solani* and *Sclerotinea rolscii* (Ganeshan and Manoj Kumar, 2005) and is known to stimulate plant growth by producing growth-promoting substances such as Indole-3-acetic acid and siderophores (Sah et al., 2021). However, their poor survivability in dynamic soil ecosystems, host specificity, etc. (Shah et al., 2021) requires enriching a catalog of plant growth-promoting strains. Therefore, identifying dual players from the host native niches will bestow the advantage of natural colonization and prepare a stage for its utilization up to its full potential. Hereby, the present study was designed to explore/elucidate the wheat rhizosphere microbial world to identify potential biocontrol agents with plant growth-promoting potential to increase wheat crop yield.

## Methods

### Isolation of wheat rhizosphere microbes

Rhizospheric soil was collected from wheat plants grown in an experimental field at Maharshi Dayanand University Rohtak (28° 52’ 44’’ NL and 76° 37’ 19’’ EL), Haryana, India. A measurement of 5.0g of soil was suspended in 20 ml ultrapure sterile water to perform physiochemical analysis. Furthermore, soil suspension was serially diluted up to 10^-8^. A measurement of 0.1ml of each dilution was spread evenly on self-devised minimal media A (pH 7.2) [Urea (200mg), calcium phosphate (250mg), Ferrous sulfate (20mg), Synthetic Sea salt (200mg), Pectin (50mg), Inulin (50mg), Starch (50mg), Sorbitol (50mg), Carboxyl methyl cellulose (50mg), and Ammonium sulfate (50mg) dissolved in 100ml distilled water]. Culture plates were incubated at 16°C, 25°C, and 37°C to isolate diverse microbes. The bacterial growth was observed for 48 hours, and morphologically diverse microbial colonies were sub-cultured at 37°C to achieve their pure cultures.

### Screening of rhizosphere isolates for antifungal potential

Isolated bacterial cultures were screened to assess their antifungal activity using a disc diffusion assay (Balouiri et al., 2016). Wheat rhizosphere microbes, *Rhizoctonia solani*, and *Fusarium oxysporum* were grown in LB and YEPD broth, respectively, with continuous shaking at 200 rpm for 24 hours at 28°C. A measurement of 0.1 ml of 1.0 OD_600nm_ overnight-grown fungal culture was spread evenly on PDA plates in sterile conditions, discs were placed at the center of the plates, and 50µl of the overnight-grown bacterial culture (A600nm: 1.0) was applied to the disc, followed by incubation at 28°C for 48 hours. The fungal growth inhibition was checked by observing the presence of the growth inhibition zone (Balouiri et al., 2016).

### Molecular, physiological, and biochemical characterization of microbial isolate BCM

Gram staining of biocontrol microbe was performed with a gram staining kit (K001-1KT, Himedia). Growth of biocontrol microbe was observed at different pHs (3, 4, 5, 7, 8, 9, 10, 11, and 12) and temperatures (10°C, 15°C, 20°C, 25°C, 30°C, 35°C, 40°C, 45°C, 50°C, 55°C, and 60°C) to identify its optimal growth conditions. Its growth pattern was observed in LB broth for 48 hrs at 37°C with constant shaking at 200rpm to check its doubling time (Wang et al., 2015). Substrate utilization preference of the identified microbe was assessed with a Hi-carbo kit (Himedia, KB009A-1KT, KB009B-1KT, and KB009C-1KT). Biochemical properties of the identified strain were performed using assays for amylase (Swain et al., 2006), catalase (Iwase et al., 2013), pectinase (Oumer and Abate, 2018), cellulase (Kasana et al., 2008), esterase (Ramnath et al., 2017), and protease (Vijayaraghavan et al., 2017). The antibiotic susceptibility of the microbial isolate was assessed using a Combi IV kit (Himedia, OD023) and G-VI-plus (Himedia, OD034). The stress response physiology of the identified strain was assessed by performing assays for salt stress tolerance, metal stress tolerance, and oxidative stress (Yadav et al., 2023). DNA was extracted from the microbial isolate using the alkali lysis method (Chauhan et al., 2009). The qualitative and quantitative analysis of the DNA was performed with agarose gel electrophoresis and Qubit HS DNA estimation kits (Invitrogen, USA), respectively. The 16S rRNA gene was amplified and sequenced to decode its taxonomic affiliation using a standardized methodology (Yadav et al., 2023).

### Genome characterization and comparative genomics

*Stenotrophomonas maltophilia* BCM was sequenced using Illumina MiSeq using Nextera XT DNA Library Prep kit. Raw reads were quality checked using FASTQC v0.11.9 (http://www.bioinformatics.babraham.ac.uk/projects/fastqc) and fastQ Validator v0.1.1 (https://github.com/statgen/fastQValidator). Contaminated reads were removed to get the corrected reads. The SPAdes v3.15.1 assembler was used for the *de-novo* assembly. Further, BUSCO v5.0.0 assessment tools were used with the latest bacterial orthologue catalog (bacteria_odb10) for analyzing the completeness of a set of predicted genes in bacterial genome assemblies. Assembled contigs were used for functional annotation via PROKKA. SSU rRNA gene was extracted, and the BLASTn was performed to identify the taxonomic affiliation of BCM. Database homologs with more than 97% 16S rRNA gene similarity were chosen for comparative analysis. Genomes were downloaded from the NCBI web server and annotated via PROKKA (Seemann, 2014). J-species software (http://jspecies.ribohost.com/jspeciesws/) assessed the genome level similarity using average nucleotide identity and tetra-correlation values. CRISPR/Casin genome was identified using the CRISPR identifier. Antibiotic resistance genes using CARD identifier were assembled and contigs were used to draw a circular genomic map via the Proksee tool (https://proksee.ca/). The antibiotic resistance, metal/metalloid resistance, and oxidative stress resistance protein features were identified using rapid annotation using a subsystem technology (RAST) server (https://rast.nmpdr.org/rast.cgi?page=Jobs). The genome was checked for pathogenesis with the Island Viewer 4 with the default parameters. Phylogenomic characterization of *Stenotrophomonas maltophilia* BCM and shortlisted strains were plotted using roary_plots.py v0.1.0 (https://github.com/sanger-pathogens/Roary/blob/master/contrib/roary_plots/roary_plots.py). The core multiple sequence alignments were used to infer the phylogenomic tree using FastTree v2.1.10 (Price et al., 2010).

### Assessment of antifungal and antibiofilm activity of *Stenotrophomonas maltophilia* BCM

The biocontrol potential of *Stenotrophomonas maltophilia* BCM was assessed against *Rhizoctonia solani* and *Fusarium oxysporum* in terms of their effect on seed germination efficiency, root and shoot length of wheat plantlets (Moraes et al., 2014), and alpha-amylase activity (Singh and Kayastha, 2014). Biofilm inhibition activity of *Stenotrophomonas maltophilia* BCM was checked against *Chromobacterium violaceum.* Anti-biofilm activity was calculated by estimating the amount of violacein production by *Chromobacterium violaceum* in the presence of *Stenotrophomonas maltophilia* BCM (Adeyemo et al., 2022).

### Role of *Stenotrophomonas maltophilia* BCM on seed germination under salt stress conditions

Wheat seed germination assay was performed in the presence of *Stenotrophomonas maltophilia* BCM. Seeds were initially soaked in overnight-grown microbial culture corresponding to 10^11^ cells/ml containing different concentrations of NaCl ranging from 0.0 M to 1.0 M for 16 hours at 37°C, while control seeds were soaked directly at different concentrations of NaCl ranging from 0 to 1M for 16 hours at 37°C. Seeds were finally wrapped in germination sheets, inserted in 50 ml culture tubes containing 5 ml Hoagland solution, and incubated for 7 days in the dark at room temperature. Seed germination percentage, alpha-amylase activity, and root and shoot length were measured after the incubation (Singh and Kayastha, 2014).

### Bio-fertilizer potential of *Stenotrophomonas maltophilia* BCM

*Stenotrophomonas maltophilia* BCM were screened for nitrate reductase activity (Kim and Seo, 2018), auxin production (Ehmann, 1977), ammonia production (Bhattacharyya et al., 2020), and siderophore biosynthesis (Himpsl and Mobley, 2019) for the assessment of their bio-fertilization potential.

## Results

### Isolation and screening of microbes with biocontrol potential

The wheat rhizospheric soil from where the microbe was isolated has been collected and identified to have 7.3 pH, 22.6˚C temperature, and 11.5 ± 1.10% moisture content. Twelve morphologically diverse microbes were purified from the wheat rhizosphere, of which three showed antifungal activity against at least one fungal strain. Only the BCM strain was found to show activity against both fungal strains and was used for further study. Antifungal activity assay showed that the microbial isolate BCM led to a growth inhibition zone of 17 ± 0.57 mm and 15 ± 0.57735 mm against *Rhizoctonia solani* and *Fusarium oxysporum*, respectively. These results indicate that microbial isolate BCM harbors the potential to develop an efficient biocontrol agent.

### Taxonomic, physiological, and biochemical characterization of microbial isolate BCM

**Figure 1:**
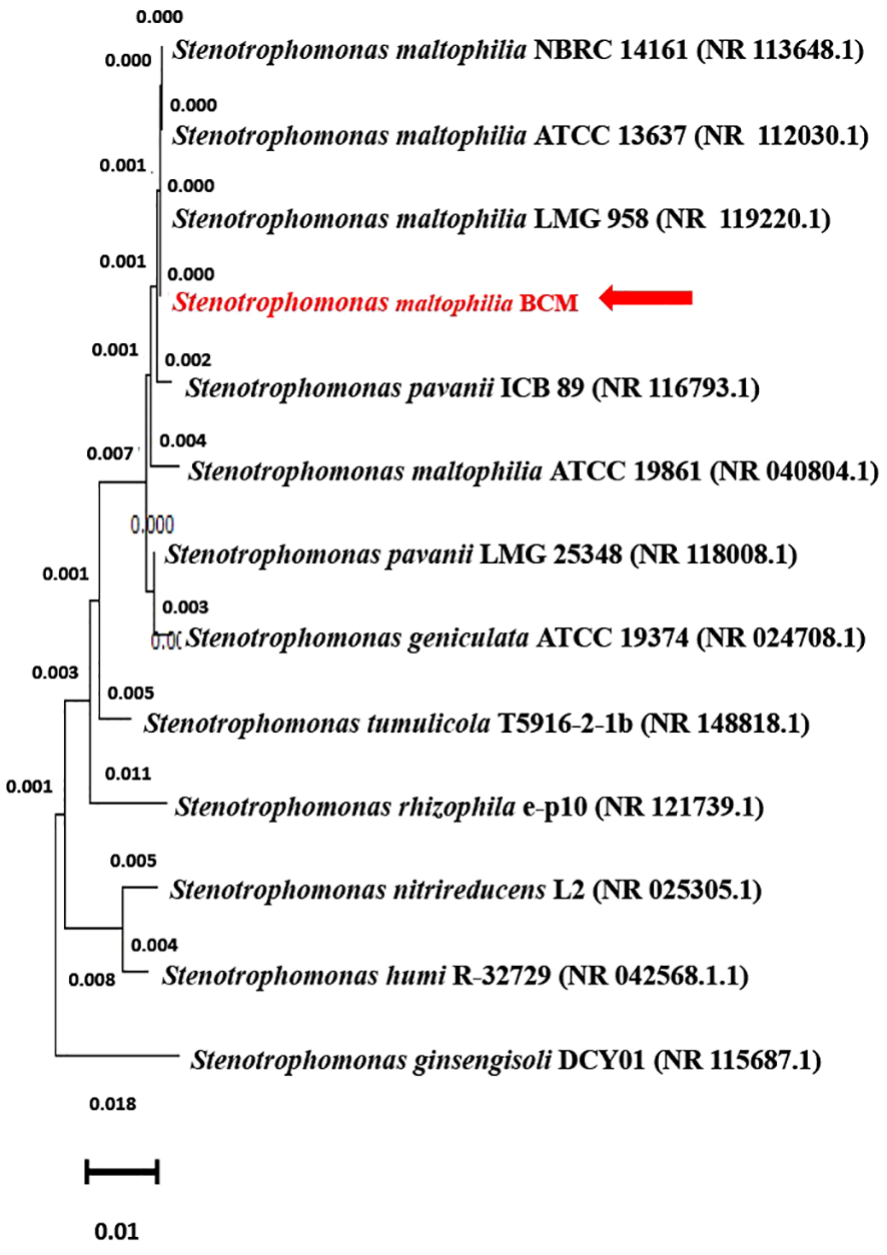
Phylogenetic affiliation of *Stenotrophomonas maltophilia* BCM with the other *Stenotrophomonas* species. The phylogenetic tree was constructed with the Neighbor-joining method of phylogenetics with 1000 bootstrap replications using MEGA-X software. The 16S rRNA gene sequence of *Stenotrophomonas ginsengisoli* DCY01 was represented as out-group.

**Figure 2:**
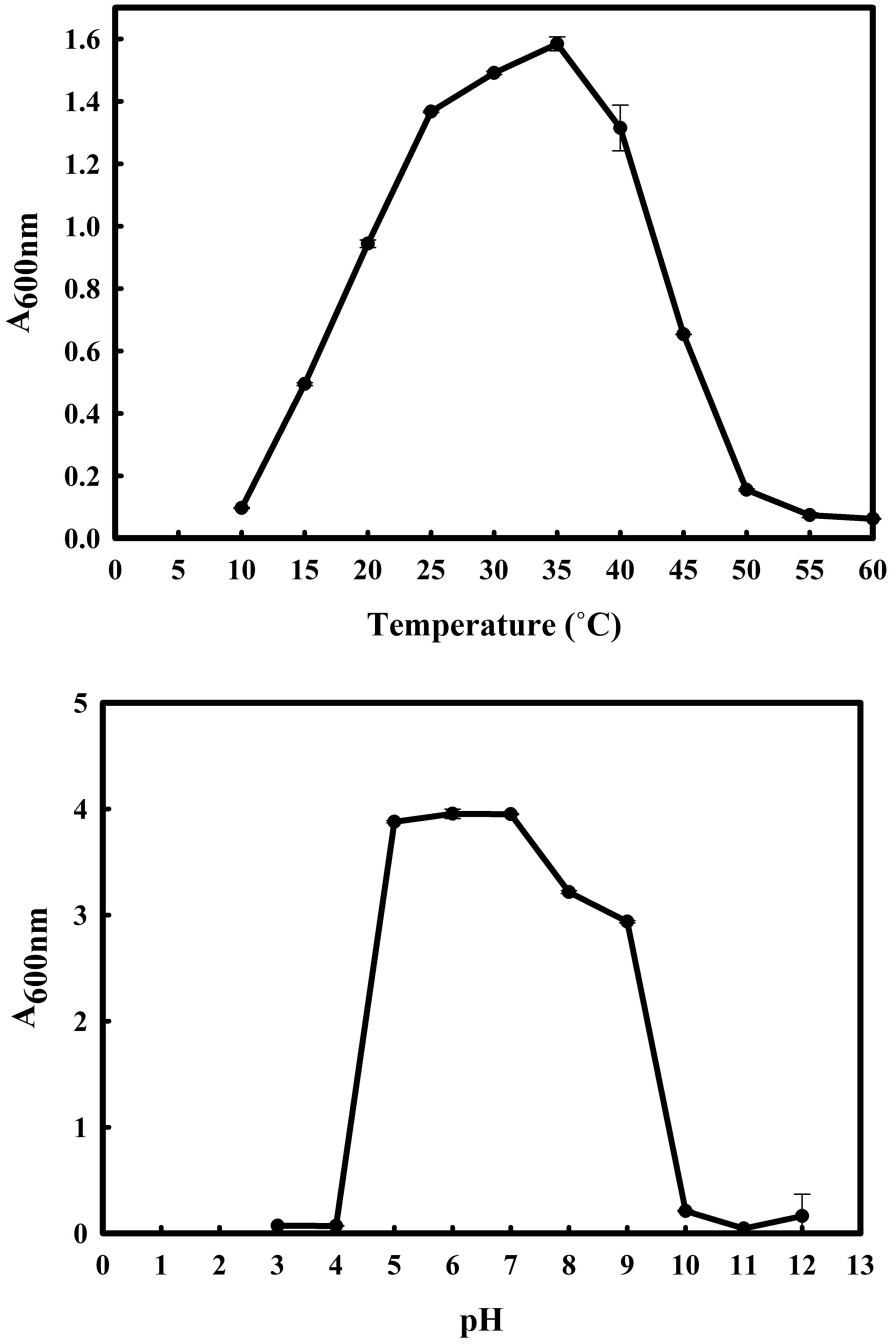
Growth profile of *Stenotrophomonas metophilia BCM* under diverse temperatures (10°C to 60°C with an interval of 5°C) and pH (from 3 to 12 with an interval of one pH unit) conditions. *Stenotrophomonas maltophilia BCM* growth was observed in LB broth after 16 hrs of uninterrupted growth at respective temperature and pH conditions with constant shaking at 200 rotations per minute (rpm). The experiments were carried out in triplicates, and growth was observed by reading the absorbance of the culture at 600nm. Plotted values are the mean of triplicates along with the observed standard deviation.

**Figure 3:**
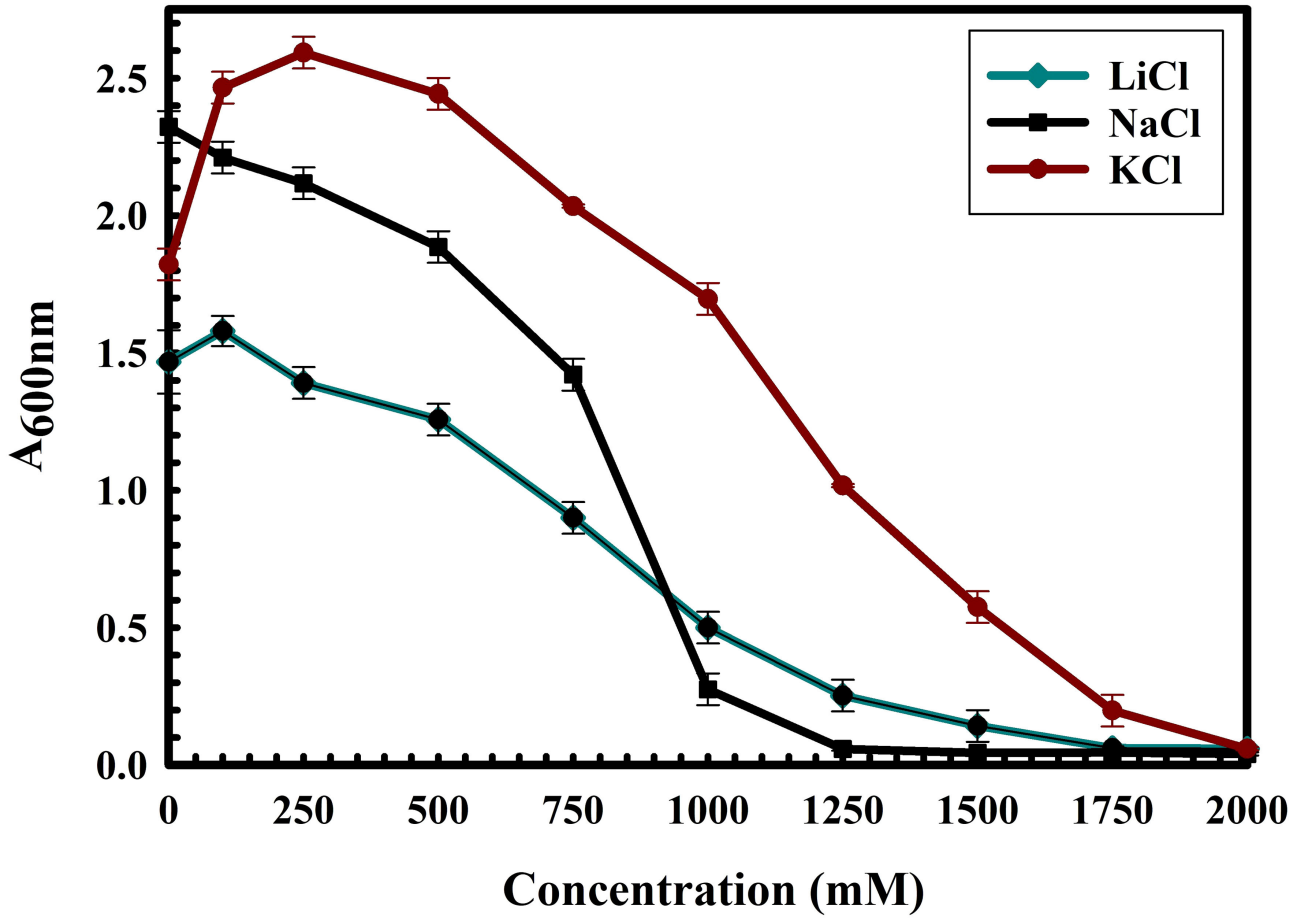
Growth profile of *Stenotrophomonas maltophilia BCM* in the presence of NaCl, KCl, and LiCl. *Stenotrophomonas maltophilia BCM* growth was observed in salt-supplemented LB broth after 16 hrs of uninterrupted growth at 37°C with constant shaking at 200 rpm. The experiments were carried out in triplicates, and growth was observed by reading the absorbance of the culture at 600nm. Values plotted here are the triplicates’ mean and the observed standard deviation.

**Figure 4:**
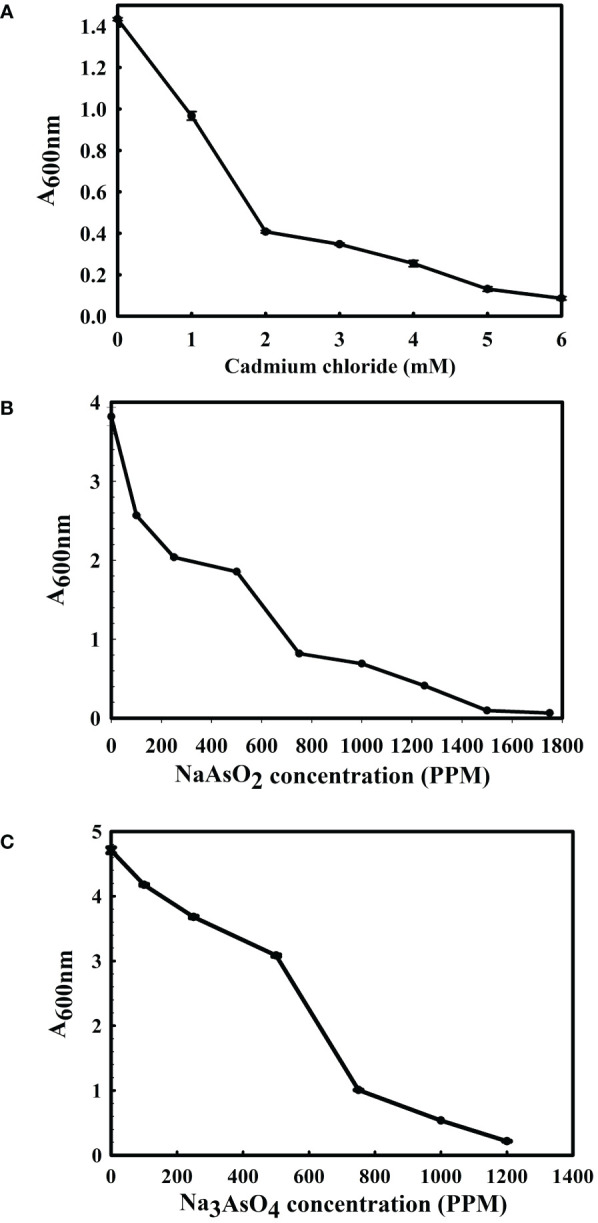
Growth profile of *Stenotrophomonas maltophilia* in the presence of cadmium chloride **(A)**, sodium arsenite **(B)**, and sodium arsenate **(C)**. *Stenotrophomonas maltophilia BCM* growth was observed in metal/metalloid-supplemented LB broth after 16 hrs of uninterrupted growth at 37°C with constant shaking at 200 rpm. The experiments were carried out in triplicates, and growth was observed by reading the absorbance of the culture at 600nm. Values plotted here are the triplicates’ mean and the observed standard deviation.

**Figure 5:**
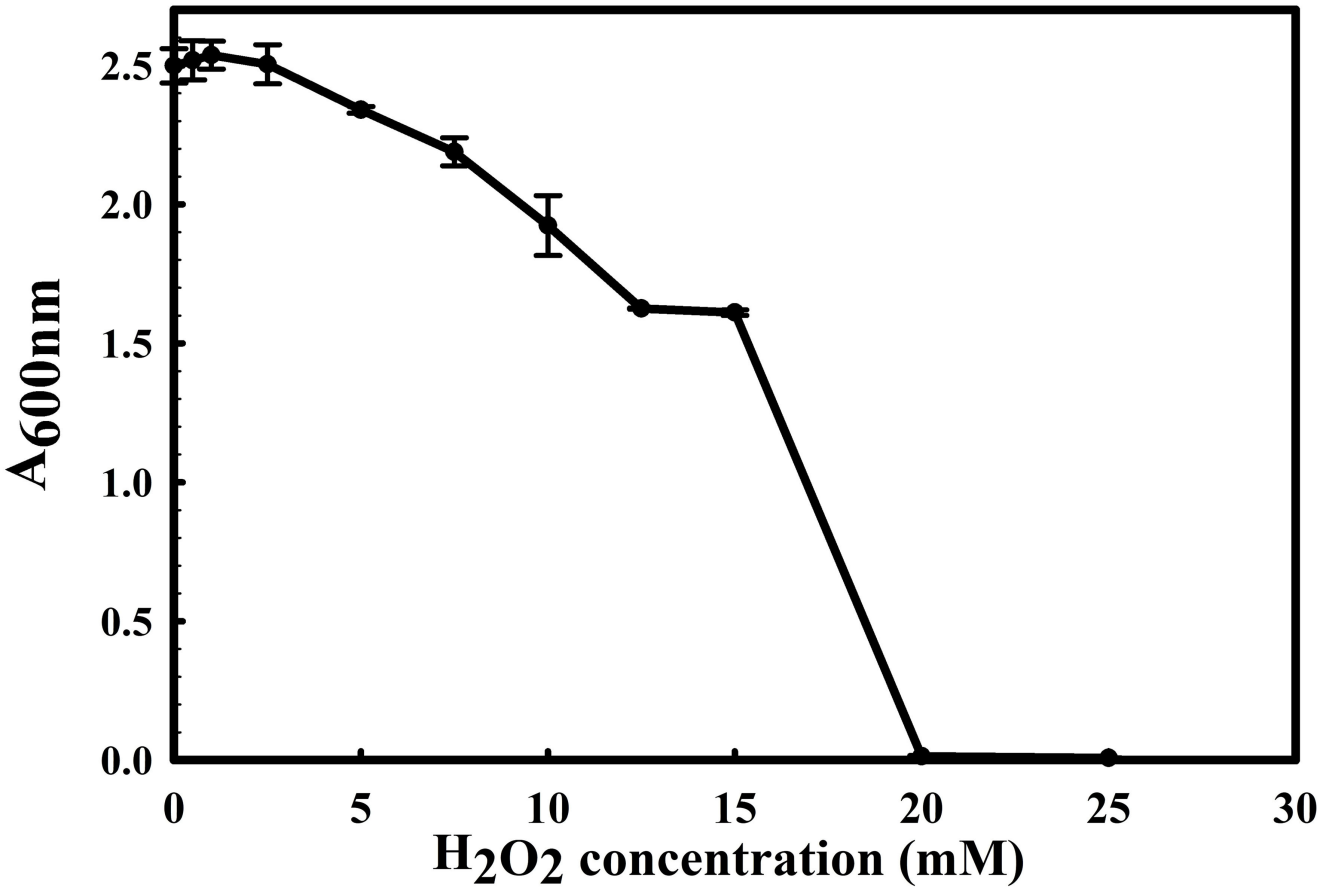
Growth profile of *Stenotrophomonas maltophilia* BCM in the presence of NaCl , KCl, and LiCl. *Stenotrophomonas maltophilia* BCM growth was observed in salt-supplemented LB broth after 16 hrs of uninterrupted growth at 37°C with constant shaking at 200 rpm. The experiments were carried out in triplicates, and growth was observed by reading the absorbance of the culture at 600nm. Values plotted here are the triplicates' mean and the observed standard deviation.

The 16S rRNA gene of the microbial isolate BCM shared 99.48% homology with *Stenotrophomonas maltophilia* LMG 25348 in the NCBI 16S rRNA gene database, indicating it as a species of *Stenotrophomonas maltophilia.* The16S rRNA gene-based phylogenetic analysis also confirms similar observations (**Figure 1**). Based on taxonomic and phylogenetic observations, microbial isolate BCM was labeled *Stenotrophomonas maltophilia* BCM for downstream analysis. Microscopic investigation indicated *Stenotrophomonas maltophilia* BCM as a gram-negative, rod-shaped, and motile bacterium. *Stenotrophomonas maltophilia* BCM showed optimum growth at pH 7.0 and 35˚C (**Figure 2**). Growth pattern analysis indicates that it attains a log phase of growth after 15 hours and has a doubling time of around 67.8 minutes (**Supplementary Figure SF1**). *Stenotrophomonas maltophilia* BCM showed growth of 0.493 O.D. at 600nm when grown anaerobically for 24 hrs at 37°C, indicating its facultative anaerobic nature. *Stenotrophomonas maltophilia* BCM was positive for amylase, esterase, lipase, protease, and catalase activity. Substrate utilization assay of *Stenotrophomonas maltophilia* BCM indicates that its substrate utilization profile is similar to other *Stenotrophomonas maltophilia* species (**Supplementary Table S1**). Antibiotic susceptibility assay suggests it is resistant toward bacitracin, cephalothin, erythromycin, novobiocin, oxytetracycline, ceftazidime, cefotaxime, and ofloxacin antibiotics while showing sensitivity toward nation, lincomycin, claxon, and amikacin. The antibiotic resistance profile of *Stenotrophomonas maltophilia* BCM was similar to other *Stenotrophomonas maltophilia* species while overlapping with *Stenotrophomonas maltophilia* smyn44 (**Supplementary Table S2**). Similar biochemical, substrate utilization, and antibiotic resistance profiles of *Stenotrophomonas maltophilia* BCM to other *Stenotrophomonas maltophilia* species strengthen the 16S rRNA gene-based taxonomic observations. Stress response physiology assays indicate that it can successfully grow in the presence of salts [up to 5.85% NaCl (w/v), 8.9% KCl (w/v), and 4.2% LiCl (w/v)] (**Figure 3**), metals [up to 0.1% Na_3_AsO_4_ (w/v), 0.12% NaAsO_2_ (w/v), and 0.54% CdCl_2_(w/v)] (**Figure 4**), oxidizing agents [up to 5.96% (v/v) H_2_O_2_] (**Figure 5**), as observed for other *Stenotrophomonas* sp. (**Supplementary Table S3**).

### Genomic characterization of *Stenotrophomonas maltophilia* BCM

**Figure 6:**
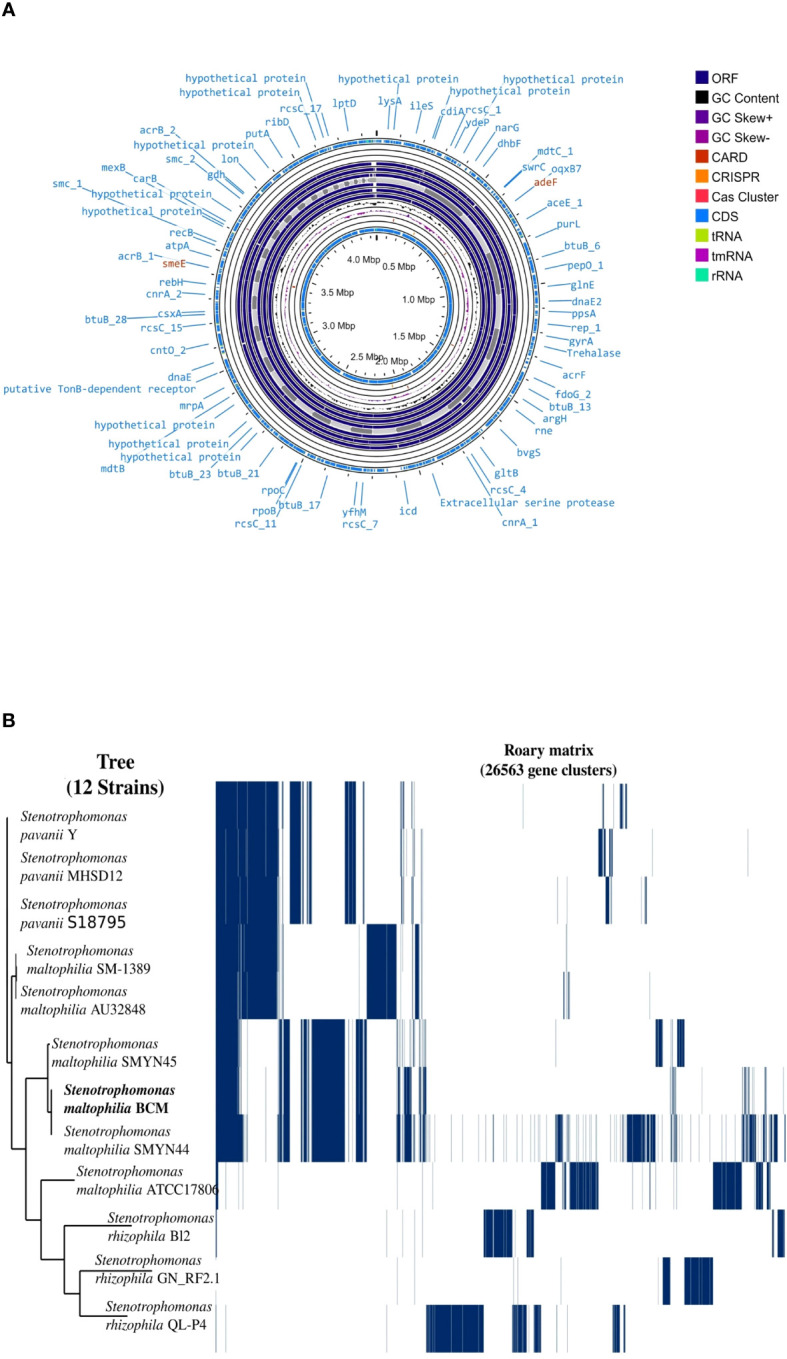
Genome map **(A)** and phylogenomic profile **(B)** of the *Stenotrophomonas maltophilia* BCM. The circular genome map **(A)** was drawn using the Proksee online tool (https://proksee.ca. The phylogenomic tree **(B)** was constructed with the FastTree v2.1.10 tool via Roary.

Genome sequencing of *Stenotrophomonas maltophilia* BCM resulted in the generation of 551495 paired-end raw reads. *Stenotrophomonas maltophilia* BCM genome was assembled into 447 contigs, accounting for a total 4519592 bp size, with 66.5% GC content and N50 length of 139100bp (**Supplementary Table S4**). Functional annotation of the genome identified 3949 protein-coding, 7 rRNA, 74 tRNA, and 1 tmRNA gene (**Figure 6A**). Average nucleotide identity (ANI) was performed to check the relationship of microbe at the genomic level. The average ANI among different species of *Stenotrophomonas* was 77-99% toward the lower end of the 77-100% spectrum, suggesting significant interspecific genomic variations. Furthermore, the ANI score of *Stenotrophomonas maltophilia* BCM with *Stenotrophomonas maltophilia* smyn44 was 99.57, while it was comparatively higher compared with other species members (**Supplementary Table S5**). The affiliation of *Stenotrophomonas maltophilia* BCM as a member of *Stenotrophomonas maltophilia* species was further confirmed using terra correlation. *Stenotrophomonas maltophilia* BCM has been awarded a 0.9996 z-score against *Stenotrophomonas maltophilia* AU12-09 during terra-correlation, confirming its similarity with *Stenotrophomonas maltophilia*. Other *Stenotrophomonas* species exhibited good similarity (z-score ∼ 0.93-0.99) (**Supplementary Table S6**). The matrix generated using the Roary tool showed the comprehensive nature of the genome in which the microbial isolate showed the highest similarity with *Stenotrophomonas maltophilia* smyn44 (**Figure 6B**). The genomic matrix also revealed that all *Stenotrophomonas* genomes share only a few numbers of genes as their core genome. Shell and cloud genome collectively forms the central part of the genomes. *Stenotrophomonas maltophilia* BCM genome has neither a pathogenic gene/island nor any virulence-related genes, indicating its non-pathogenic nature. In addition to the genes for antimicrobial activity (**Table 1**), *Stenotrophomonas maltophilia* BCM genome harbors genes encoding proteins for plant growth promotion activities like auxin biosynthesis, nitrogen assimilation, siderophore biosynthesis, and phosphate solubilization (**Table 2**). *Stenotrophomonas maltophilia* BCM genome also harbors genes responsible for arsenic resistance, oxidative stress tolerance, metal stress tolerance, and salt tolerance (**Supplementary Table S7**), explaining its stress response physiology. The 24 CAZymes clusters within its genome also justify its diverse carbohydrate utilization profile (**Supplementary Table S8**). Additionally, its genome encodes various hydrolases, some of which might extend anti-pathogenic behavior to the host (**Supplementary Table S9**).

**Table 1:** Genetic features involved in *Stenotrophomonas maltophilia* BCM genome involved in biocontrolling activity.

CDS start Position in the genome	CDS stop position in the genome	Strand	Function
A. Phenazine biosynthesis and resistance protein			
11738	10860	–	Phenazine biosynthesis protein PhzF
12685	11810	–	Phenazine biosynthesis protein PhzF
18001	16053	–	Phenazine antibiotic resistance protein EhpR
B. Bacteriocin resistance protein			
37151	36447	–	Bacteriocin resistance protein
C. Chitinase			
181286	180096	–	Chitinase
61552	63651	+	Chitinase

**Table 2:** Genetic features identified within *Stenotrophomonas maltophilia* BCM genome encoding various proteins involved in nutrient assimilation and solubilization.

CDS start position in the genome	CDS stop position in the genome	Strand	Function
A. Nitrogen transport and regulation			
69574	69969	+	Nitrogen regulatory protein P-II, GlnK
94361	95422	+	Nitrogen regulation protein NtrB (EC 2.7.13.3)
95415	96863	+	Nitrogen regulation protein NR(I), GlnG (=NtrC)
26694	25342	–	nitrogen regulation protein NtrY, putative
348	10	–	Nitrogen regulatory protein P-II, GlnK
36588	37013	+	PTS IIA-like nitrogen-regulatory protein PtsN
145605	147023	+	Nitrate/nitrite transporter NarK/U
147096	150839	+	Respiratory nitrate reductase alpha chain (EC 1.7.99.4)
150839	152383	+	Respiratory nitrate reductase beta chain (EC 1.7.99.4)
152383	153063	+	Respiratory nitrate reductase delta chain (EC 1.7.99.4)
153060	153767	+	Respiratory nitrate reductase gamma chain (EC 1.7.99.4)
B. IAA biosynthesis			
36312	35470	–	Indole-3-glycerol phosphate synthase (EC 4.1.1.48)
C. Phosphate regulation, transport, and solubilization			
189720	188125	–	Alkaline phosphatase
23081	23770	+	Phosphate regulon transcriptional regulatory protein PhoB (SphR)
23877	25208	+	Phosphate regulon sensor protein PhoR (SphS) (EC 2.7.13.3)
39099	38392	–	Phosphate transport system regulatory protein PhoU
40006	39176	–	Phosphate ABC transporter, ATP-binding protein PstB (TC 3.A.1.7.1)
40889	40026	–	Phosphate ABC transporter, permease protein PstA (TC 3.A.1.7.1)
41857	40889	–	Phosphate ABC transporter, permease protein PstC (TC 3.A.1.7.1)
43028	41940	–	Phosphate ABC transporter, substrate-binding protein PstS (TC 3.A.1.7.1)
44453	43437	–	Phosphate ABC transporter, substrate-binding protein PstS (TC 3.A.1.7.1)
45887	44676	–	Phosphate/pyrophosphate-specific outer membrane porinOprP/OprO
D. Siderophore Biosynthesis			
83949	81742	–	Putative OMR family iron-siderophore receptor precursor
1	546	+	TonB-dependent siderophore receptor
77730	78392	+	Ferric siderophore transport system, biopolymer transport protein ExbB
104477	105670	+	Isochorismate synthase (EC 5.4.4.2) @ Isochorismate synthase (EC 5.4.4.2) of siderophore biosynthesis
105667	107319	+	2,3-dihydroxybenzoate-AMP ligase (EC 2.7.7.58) of siderophore biosynthesis
107319	107951	+	Isochorismatase (EC 3.3.2.1) of siderophore biosynthesis
108205	112095	+	Siderophore biosynthesis non-ribosomal peptide synthetase modules
112086	112844	+	2,3-dihydro-2,3-dihydroxybenzoate dehydrogenase (EC 1.3.1.28) of siderophore biosynthesis
202780	203736	+	Iron siderophore sensor protein
203920	207042	+	Iron siderophore receptor protein

Several proteins were identified as essential for effective colonization in plant rhizosphere (Kumar et al., 2023). An in-depth analysis of *the Stenotrophomonas maltophilia* BCM genome identifies *the* presence of genes encoding proteins for the synthesis of Type 1 and IV pili, exopolysaccharide (**Table 3**) essential for plant surface adhesion, auto-aggregation, and early biofilm formation (Kumar et al., 2023).

**Table 3:** Genetic features within the genome encoding proteins for *Stenotrophomonas maltophilia* BCM colonization in wheat rhizosphere.

CDS start position in the genome	CDS stop position in the genome	Strand	Function
A. Pili formation Protein			
36034	35504	–	Type IV pili signal transduction protein PilI
61031	61546	+	Type IV fimbrial biogenesis protein FimT
61543	62040	+	Type IV fimbrial biogenesis protein PilV
62049	63206	+	Type IV fimbrial biogenesis protein PilW
63212	63733	+	Type IV fimbrial biogenesis protein PilX
63747	67514	+	Type IV fimbrial biogenesis protein PilY1
67538	67945	+	Type IV pilus biogenesis protein PilE
B. Mannose-6-phosphate isomerase			
67945	68009	+	Mannose-6-phosphate isomerase
C. EPS biosynthesis			
19212	19874	+	Exopolysaccharide synthesis ExoD

### Assessment of the antifungal potential of *Stenotrophomonas maltophilia* BCM

**Figure 7:**
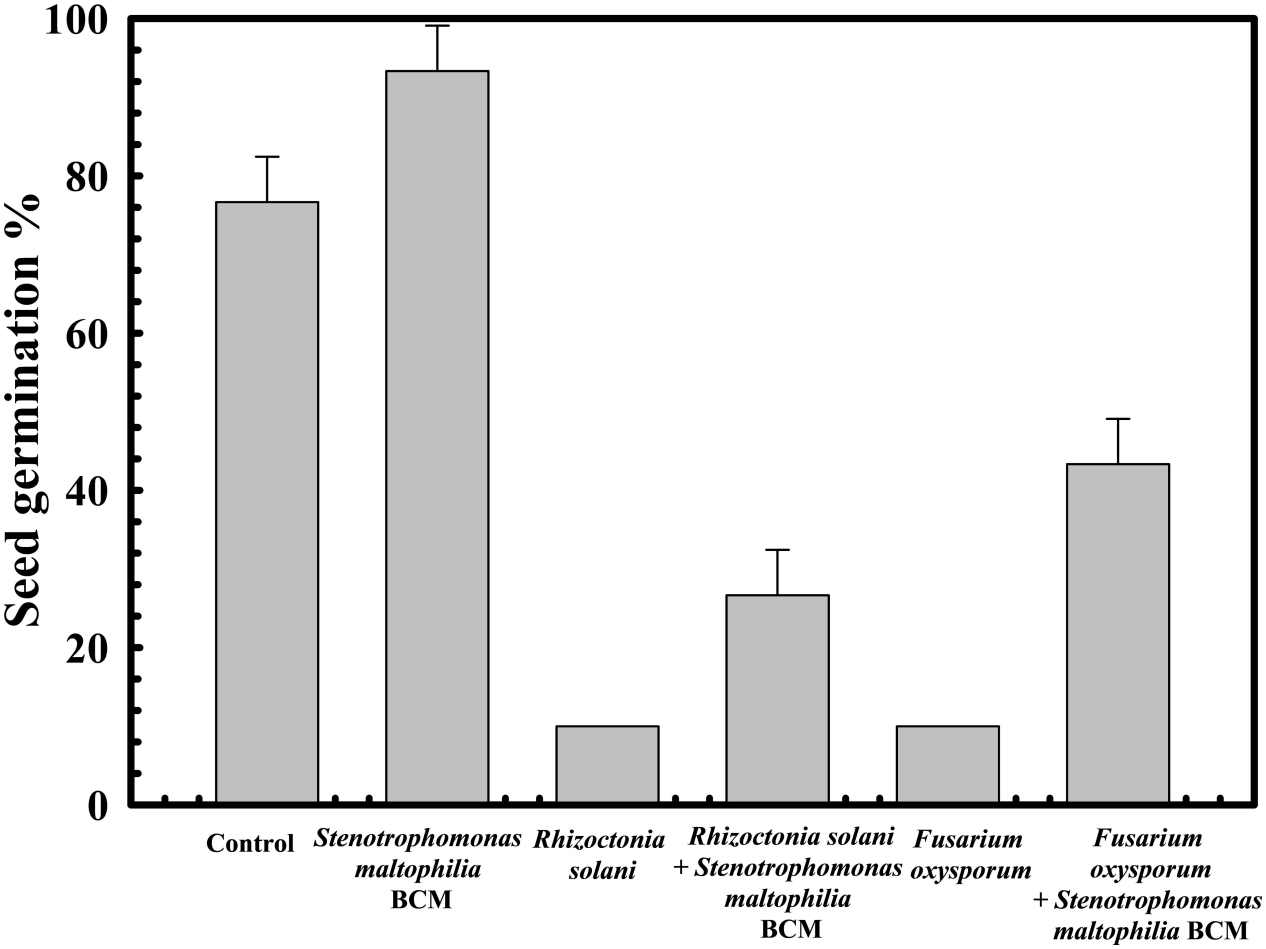
Impact of *Stenotrophomonas maltophilia* BCM on the wheat seed germination percentage under biotic stress. Seeds were pre-inoculated with 2× 10^8^ CFU/ml of test organisms (*S. maltophilia* BCM and phytopathogenic fungal strains as per experimental conditions) for 16 hours before seed germination experiments. All assays were carried out in triplicates.

**Figure 8:**
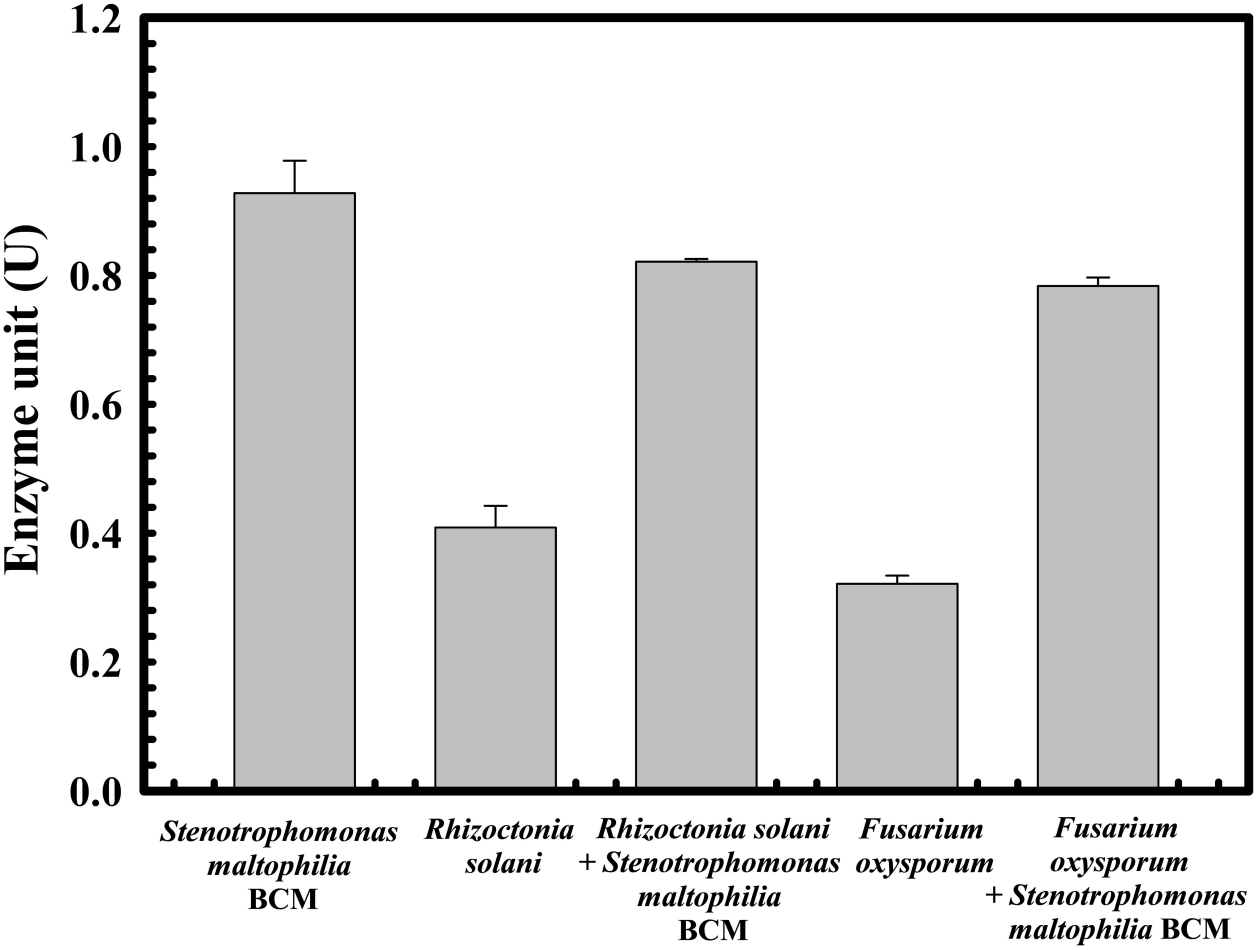
Impact of *Stenotrophomonas maltophilia* BCM on the alpha-amylase activity in wheat seeds during germination under biotic stress. Seeds were pre-inoculated with 2× 10^8^ CFU/ml of test organisms (*S. maltophilia* BCM and phytopathogenic fungal strains as per experimental conditions) for 16 hours before alpha-amylase activity assays. Plotted values are the mean of triplicates along with the observed standard deviation.

The protective effect of *Stenotrophomonas maltophilia* BCM on wheat seed germination was assessed. A 10% ± 1 and 5% ± 0.57735 seed germination was observed in the presence of *Rhizoctonia solani* and *Fusarium oxysporum*, respectively. Seeds’ pre-treatment with *Stenotrophomonas maltophilia* BCM showed a germination efficiency of 75.33 ± 0.57735% and 87.66 ± 0.57705% in the presence of *Rhizoctonia solani* and *Fusarium oxysporum*, respectively (**Figure 7**). *Stenotrophomonas maltophilia* BCM was observed to increase ~750 and ~1753-fold seed germination in the presence of *Rhizoctonia solani* and *Fusarium oxysporum*, respectively. These results strongly indicate the potential biocontrol behavior of *Stenotrophomonas maltophilia* BCM*. Stenotrophomonas maltophilia* BCM enhanced seed germination in the presence of phytopathogens and significantly improved seed germination in reference to the control (P=0.024) (**Figure 7**). *Rhizoctonia solani* and *Fusarium oxysporum* were also found to significantly reduce (P=0.0001 and P=0.0018) alpha-amylase activity in wheat seeds to 0.42 IU and 0.22 IU, respectively. Seeds’ pre-treatment with *Stenotrophomonas maltophilia* BCM significantly increased alpha-amylase activity (P=0.000136 and P=0.0000262) in the presence of *Rhizoctonia solani* and *Fusarium oxysporum*, respectively (**Figure 8**). The significant improvement of alpha-amylase activity in wheat seeds after pre-treatment with *Stenotrophomonas maltophilia* BCM could be a possible reason for enhanced seed germination.

**Figure 9:**
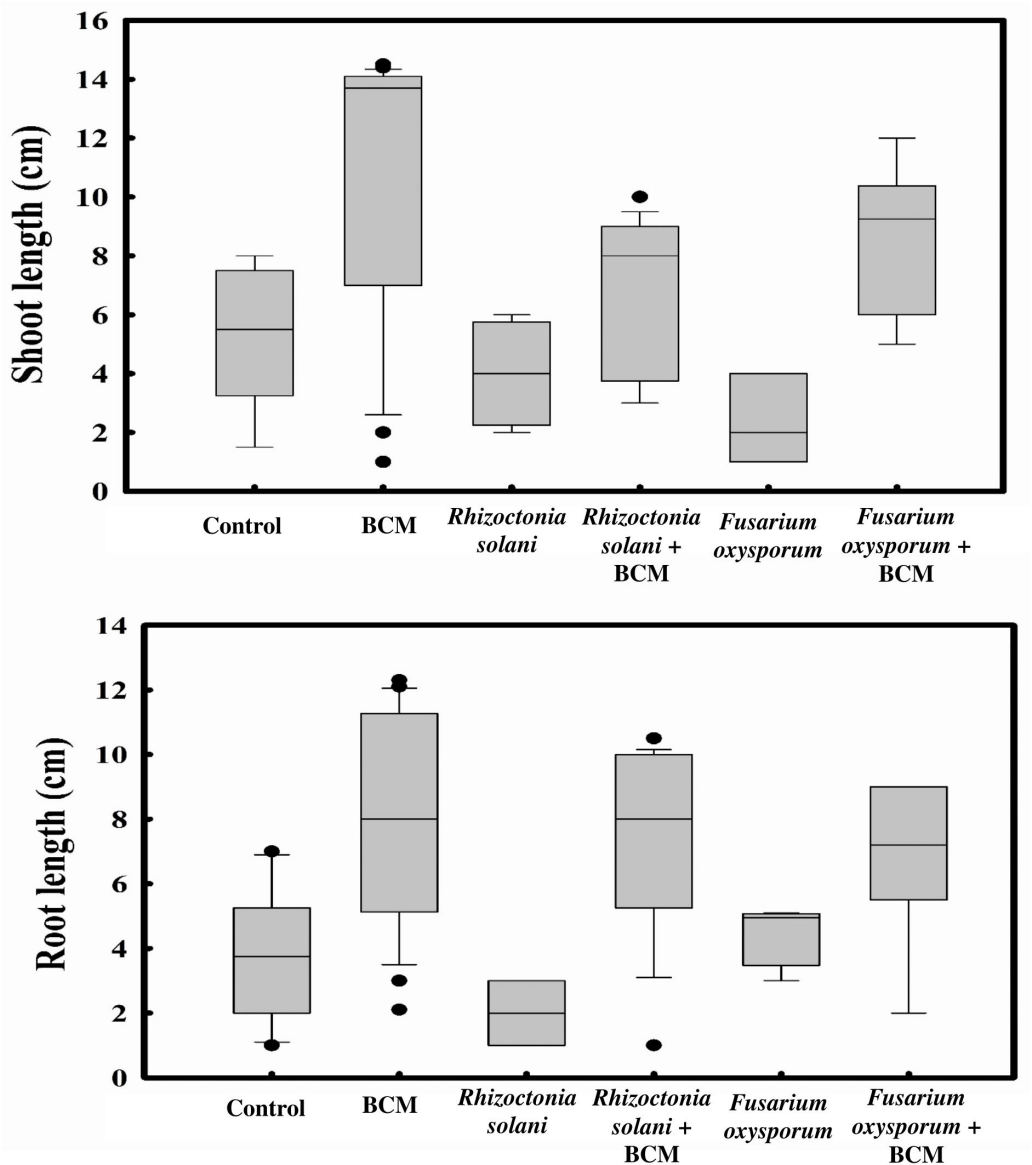
Impact of *Stenotrophomonas maltophilia* BCM on root and shoot length of WC-306 plantlets under biotic stress. Seeds were pre-inoculated with 2× 10^8^ CFU/ml of test organisms (*S. maltophilia* BCM and phytopathogenic fungal strains as per experimental conditions) for 16 hours before seedling growth experiments. Plotted values are the mean of triplicates along with the observed standard deviation.

Pre-treatment of seeds with *Stenotrophomonas maltophilia* BCM not only enhanced seed germination but also improved the growth of wheat plantlets. Wheat seeds pre-treated with *Stenotrophomonas maltophilia* BCM showed a significantly enhanced shoot length (P=0.027) and root length (P=0.010) compared to the untreated seeds (**Figure 9**). *Stenotrophomonas maltophilia* BCM was also found to significantly improve the root (P=0.017 and 0.039) and shoot length (P=0.020 and 0.136) of wheat plantlets infected with *Rhizoctonia solani* and *Fusarium oxysporum*, respectively. The average shoot and root length of wheat plantlets treated with *Stenotrophomonas maltophilia* BCM were significantly higher (P=0.0297 and 0.0023) compared to the control even after *Rhizoctonia solani* and *Fusarium oxysporum* exposure, indicating its biocontrol behavior. *Stenotrophomonas maltophilia* BCM also interacted with *Chromobacterium violaceum* and significantly reduced violacein production (P=0.001). This indicates the potential of *Stenotrophomonas maltophilia* BCM as a quorum-sensing inhibitor and antibiofilm activity, which is good for a bio-controlling agent.

**Figure 10:**
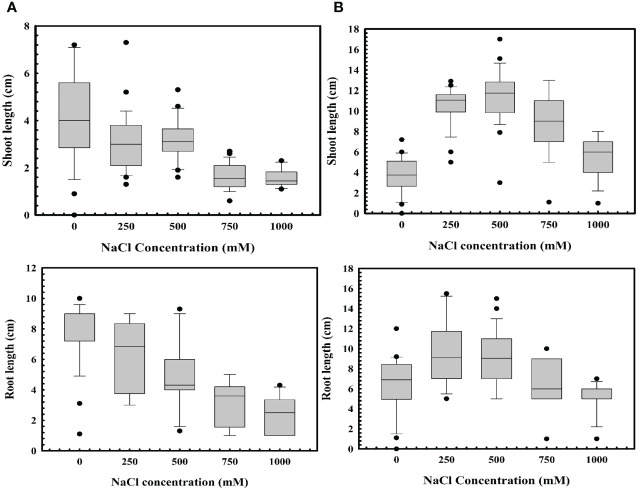
Impact of *Stenotrophomonas maltophilia* BCM on root and shoot length of WC-306 plantlets under saline stress (NaCl **(A)** and KCl **(B)**). Seeds were pre-inoculated with 2× 10^8^ CFU/ml of test organisms (*S. maltophilia* BCM and phytopathogenic fungal strains as per experimental conditions) for 16 hours before seedling growth experiments. Plotted values are the mean of triplicates along with the observed standard deviation.

*Stenotrophomonas maltophilia* BCM’s role in wheat seed germination in abiotic stress like high salinity (a key bottleneck in wheat germination and crop production) was also assessed. Increased salt concentration significantly reduced seed germination (**Figure 10A**). Seeds’ pre-treatment with *Stenotrophomonas maltophilia* BCM showed enhanced seed germination at high salinity conditions (**Figure 10B**). Pre-treatment of seeds with *Stenotrophomonas maltophilia* BCM not only enhanced seed germination but also improved the growth of wheat plantlets. Wheat seeds pre-treated with *Stenotrophomonas maltophilia* BCM showed a significantly enhanced shoot length (P=0.001) and root length (P=00.0018) compared to the untreated in high salinity conditions.

### Plant growth potential of *Stenotrophomonas maltophilia* BCM

*Stenotrophomonas maltophilia* BCM genome harbors key genes responsible for plant growth promotion activities like auxin biosynthesis, nitrogen assimilation, siderophore biosynthesis, and phosphate solubilization. *Stenotrophomonas maltophilia* BCM showed nitrate reductase activity (14IU), extracellular alkaline (0.22IU), and acid phosphatase activity (0.1 IU). It was also found to produce and secrete plant growth-promoting hormones in the surrounding environment. The presence of genes responsible for plant growth promotion and their bioactivity indicate biocontrol behavior. Stenotrophomonas maltophilia BCM also harbors plant growth-promotion activity.

## Discussion

A boost in crop production of cereals is required to ensure food security for the expanding global population (Tilman et al., 2011). In the past, applying agrochemicals has boosted agricultural yield and helped to fulfill food requirements. Continuous applications of agrochemicals negatively impact the environment and human health. These agrochemicals severely affect soil fertility, creating hurdles in enhancing crop production to ensure food security (Gamage et al., 2023). In addition, the evolution of plant pathogens for pesticide resistance, higher infectivity, and broad host range are significant challenges (Newman and Derbyshire, 2020). Sustainable agricultural practices offer solutions to ensure healthy soil ecology, limit infections, and enhance crop production (Mehmet Tuğrul, 2020). Plants harbor several microbial companions on underground and above-ground surfaces (Van Dijk et al., 2022). These micro-residents improve the host plant’s growth by enhancing nutrient assimilations, cell division, and plant reproduction and preventing the invasion of pathogens (Koza et al., 2022). Sustainable agricultural practices advocate the employment of such micro-residents to improve crop production. Identification and characterization of such candidate microbes became a quest of researchers around the globe.

Wheat is one of the prime food sources for fulfilling the hunger of most of the global population. Increases in soil salinity, reduced soil fertility, emergence of phytopathogens, and climate change threaten wheat production (Srinivas et al., 2019). There is an emergent need to identify potential microbial agents that can improve resistance toward both biotic (phytopathogens) and abiotic (salinity) stress in wheat for better crop production. In the present study, an effort was made to explore the wheat rhizosphere microbiota to identify potential microbial candidates to enhance plant growth by inhabiting phytopathogens infection, improving nutrient assimilation, and extending resistance toward various stressors.

Culture-dependent exploration of wheat rhizosphere microbiota identified 12 morphologically different bacterial isolates. Antifungal assays indicate that the isolate BCM showed effective growth inhibition of *Rhizoctonia solani* and *Fusarium oxysporum* phytopathogens. The 16S rRNA gene-based phylogenetic characterization of BCM isolate reveals suitable homology with *Stenotrophomonas maltophilia*, accordingly labeled as *Stenotrophomonas maltophilia* BCM*. Stenotrophomonas maltophilia* is a group of diazotrophic bacteria isolated from diverse habitats, predominantly from plant-associated ecosystems (Pinski et al., 2020). Rhizospheric *Stenotrophomonas maltophilia* species have the potential as biofertilizers, biocontrol agents, pesticide remediation (Kumar et al., 2023), and stress resistance (Singh and Jha, 2017). These studies strengthen the candidature of *Stenotrophomonas maltophilia* BCM as a biofertilizer and biocontrol agent. Most of this information was drawn based on experimentation with diverse plant species (Kumar et al., 2023). As a result, it is essential to understand its physiological, genomic, and plant-associated properties to understand its efficiency.

The morphological, physiological, and chemotaxonomic profile of BCM was similar to other *Stenotrophomonas maltophilia* strains, confirming 16S rRNA gene-based phylogenetic observations. Likewise, other *Stenotrophomonas* sp.*, Stenotrophomonas maltophilia* BCM, was observed to successfully thrive under diverse physicochemical (pH and temperature) and stress conditions (saline, exposure to metal/metalloid, and antibiotic). Survival in diverse environments extended the ubiquitous nature of *Stenotrophomonas* sp (Kumar et al., 2023). *Stenotrophomonas maltophilia* BCM harbors a G+C-rich genome of 4519592bps encoding a diverse array of proteins, extending metabolic robustness to the host. Average Nucleotide Identity and phylogenomic observations further validate its taxonomic affiliation. *Stenotrophomonas maltophilia* BCM encodes a diverse array of hydrolytic enzymes (CAzymes, proteases, chitinase, glucanases, and lipases), phytohormones production (IAA), nutrients (Phosphate) solubilization, and phenazine production. The presence of protein-encoding features for phosphate solubilization, siderophore production, nitrogen fixation, and phytohormone production could significantly boost plant growth (Souza et al., 2015) to act as a biofertilizer. Various *Stenotrophomonas* sp. were already characterized for the presence of these features and were placed under the category of PGPRs (Majeed et al., 2015). Chitinolytic and proteolytic enzymes could effectively hydrolyze fungal cell walls and inhibit fungal growth (Hamid et al., 2013). Phenazine is another potent antifungal compound, and phenazine-producing microbes can effectively protect plants against fungal phytopathogens (Karmegham et al., 2020). *Stenotrophomonas maltophilia* BCM genome harbors genes encoding chitinase, protease, and proteins involved in phenazine production, indicating its potential as a biocontrol agent. Type I and IV pili encoding genes are essential for adhesion, autoaggregation, and biofilm formation (Elvers et al., 2001); these proteins allow the PGPR *Stenotrophomonas* sp. to associate with plant host in the rhizosphere (Kumar et al., 2023). The presence of these genes in the *Stenotrophomonas maltophilia* BCM genome further confirms its strong interaction with wheat rhizosphere. Despite the enormous potential of *Stenotrophomonas* sp., their application for crop improvement is challenged by the pathogenic nature of *Stenotrophomonas maltophilia* (Ryan et al., 2009). Surprisingly, the *Stenotrophomonas maltophilia* BCM genome lacks gene clusters for inducing pathogenicity in animals and plants, confirming its safe application. *Stenotrophomonas maltophilia* BCM genome indicates its application as a biocontrol agent and biofertilizer; however, these properties must be validated experimentally.

*In-vitro* experiments confirm phosphate solubilization, nitrate reduction, and auxin synthesis properties of *Stenotrophomonas maltophilia* BCM. These experimental observations confirm genomic observations based on the biofertilizer potential of *Stenotrophomonas maltophilia* BCM. Additionally, *in-vitro* antifungal experiments confirm its potential as a biocontrol agent. PGPR and biocontrol behavior of *Stenotrophomonas maltophilia* BCM were further confirmed in *in-vivo* studies. Wheat seed germination experiments in the presence of phytopathogens, *Fusarium oxysporum*, and *Rhizoctonia solani* indicated that *Stenotrophomona smaltophilia* BCM effectively protects the host seedling from fungal infection. These observations support the genome-based observations indicting *Stenotrophomonas maltophilia* BCM as a biocontrol agent. *Stenotrophomonas maltophilia* BCM not only protects the wheat seedlings from fungal infection but also significantly improves seed germination percentage and plant growth in the presence and absence of phytopathogens. *Stenotrophomonas maltophilia* BCM also improved wheat seed germination percentage and seedling growth under saline conditions, indicating its potential to overcome salty conditions. Conclusively, wheat rhizosphere isolates *Stenotrophomonas maltophilia* BCM showed good PGPR and biocontrol potential under a diverse range of stresses in the study, projecting its potential application for sustainable agriculture. However, long-term analysis under field conditions is required to validate the outcomes. These analyses are essential for implanting the isolate in agricultural practices.

## Data availability statement

The whole genome sequence of *Stenotrophomonas maltophilia* BCM has been uploaded to the NCBI server with an SRA accession number SRX23009035 and Bio project accession ID PRJNA1056133.

## Author contributions

PS: Data curation, Formal analysis, Investigation, Methodology, Validation, Visualization, Writing – original draft, Writing – review & editing. RP: Writing – original draft, Writing – review & editing. NC: Data curation, Methodology, Project administration, Validation, Writing – original draft, Writing – review & editing.
