# Supplementary material for: Biofertilizer and biocontrol properties of Stenotrophomonas maltophilia BCM emphasize its potential application for sustainable agriculture
Source: Front Plant Sci. 2024 Mar 4;15:1364807. doi: 10.3389/fpls.2024.1364807 (PMC10944936; doi:10.3389/fpls.2024.1364807)

Biofertilizer and biocontrol properties of *Stenotrophomonas maltophilia* BCM emphasize its potential application for sustainable agriculture

Pinki Sharma[1], Rajesh Pandey[2], Nar Singh Chauhan[1*]

[1]Department of Biochemistry, Maharshi Dayanand University, Rohtak, Haryana, India

[2]Integrative GENomics of Host-PathogEn (INGEN-HOPE) laboratory, CSIR-Institute of Genomics and Integrative Biology (CSIR-IGIB), Mall Road, Delhi-110007, India

*Corresponding author

Nar Singh Chauhan (nschauhan@mdurohtak.ac.in)

Running Title: *Stenotrophomonas maltophilia* BCM for Sustainable Agriculture

Number of Words: 8013

Number of Figures: 10

Number of Tables: 3

Supplementary Table S1: Substrate utilization profile of *Stenotrophomonas maltophilia* BCM with other phylogenetic-related *Stenotrophomonas* species

Sr. No.	Substrate	***Stenotrophomonas Maltophilia*BCM**	***Stenotrophomonas Maltophilia*AuRed02**	***Stenotrophomonasindicatrix*WS 40**	***Stenotrophomonaspavanii*CCUG 59972**	***Stenotrophomonas rhizophilae*-p10**	***Stenotrophomonas chelatiphaga***
1	Lactose	ND	ND	ND	ND	ND	ND
2	Xylose	+	ND	ND	ND	ND	ND
3	Maltose	+	+	ND	ND	+	ND
4	Fructose	+	+	+	ND	ND	ND
5	Dextrose	+	+	+	ND	+	ND
6	Galactose	ND	ND	ND	ND	ND	ND
7	Raffinose	+	ND	ND	ND	ND	ND
8	Trehalose	+	+	ND	ND	ND	ND
9	Melibiose	+	ND	ND	ND	ND	ND
10	Sucrose	ND	+	ND	ND	ND	ND
11	L-Arabinose	ND	ND	ND	ND	+	ND
12	Mannose	ND	ND	+	ND	+	+
13	Inulin	+	+	ND	ND	ND	ND
14	Sodium gluconate	ND	ND	ND	ND	ND	ND
15	Glycerol	ND	+	ND	ND	ND	ND
16	Salicin	ND	ND	ND	ND	ND	ND
17	Dulcitol	+	ND	ND	ND	ND	ND
18	Inositol	ND	ND	ND	ND	ND	+
19	Sorbitol	ND	ND	ND	ND	ND	ND
20	Mannitol	ND	ND	+	ND	+	ND
21	Adonitol	+	ND	ND	ND	ND	ND

22	Arabitol	ND	ND	ND	ND	ND	ND
23	Erythritol	ND	ND	ND	ND	ND	ND
24	α-Methyl-D-glucoside	ND	ND	ND	ND	ND	ND
25	Rhamnose	+	ND	ND	ND	ND	ND
26	Cellobiose	ND	ND	ND	ND	ND	ND
27	Melezitose	ND	ND	ND	ND	ND	ND
28	α-Methyl-D-mannoside	ND	ND	ND	ND	ND	ND
29	Xylitol	+	ND	+	ND	ND	ND
30	ONPG	+	+	ND	ND	ND	ND
31	Esculin hydrolysis	ND	ND	ND	+	ND	ND
32	D-Arabinose	ND	ND	ND	ND	ND	ND
33	Citrate utilization	ND	ND	ND	+	ND	ND
34	Malonate utilisation	ND	ND	ND	ND	ND	ND
35	Sorbose	+	+	ND	ND	+	ND

Here ND: Not defined in literature

Supplementary Table S2: Comparative analysis of antibiotic susceptibility profile of *Stenotrophomonas maltophilia* BCM with other phylogenetic similar *Stenotrophomonas* strains.

**Sr. No.**	**Antibiotic**	***Stenotrophomonasmaltophilia* BCM**	***Stenotrophomonasmaltophilia*smyn44**	***Stenotrophomonasindicatrix***	***Stenotrophomonaspavanii***	***Stenotrophomonasrhizophila***	***Stenotrophomonas sp. SXG-1***	***Stenotrophomonaschelatiphaga***
1	Amikacin	ND	+	+	+	ND	ND	+
2	Amoxicillin	+	+	ND	+	ND	ND	ND
3	Bacitracin	+	ND	ND	ND	ND	ND	ND
4	Cephalothin	+	ND	ND	ND	ND	ND	ND
5	Erythromycin	+	+	ND	ND	ND	ND	ND
6	Novobiocin	+	+	ND	ND	ND	ND	ND
7	Oxytetracyclin	+	ND	ND	ND	ND	ND	ND
8	Vancomycin	+	+	ND	ND	ND	ND	ND
9	Ceflnaxone	ND	ND	ND	ND	ND	ND	ND
10	Ceflazidime	+	+	ND	ND	ND	ND	+
11	Cefotaxime	+	ND	+	ND	ND	ND	ND
12	Lincomycin	ND	ND	ND	ND	ND	ND	ND
13	Netillin	ND	ND	ND	ND	ND	ND	ND
14	Ofloxacin	+	ND	ND	ND	ND	+	ND

Here ND: Not defined in the literature.

Supplementary Table S3: A comparison of the growth parameters of *Stenotrophomonas maltophilia* BCM with other *Stenotrophomonas* strains

Sr no.	Microbes	pH	Temperature (˚C)	Minimum Inhibitory Concentration						
				**NaCl**	**KCl**	**LiCl**	**As(III)**	**As(V)**	**$CdCl_2$**	**H_2O_2**
1	***Stenotrophomonas maltophilia* BCM**	5-9.5	15-45	1250mM	2000 mM	700 mM	1200 PPM	1000 PPM	ND	17.5mM ,
2	*Stenotrophomonas chelatiphaga*	4-9	35°C	2000mM	1750 mM	1750 mM	2000 PPM	1000 PPM	ND	12.5mM
3	*Stenotrophomonas indicatrix*	4 to 10	25 to 40	3.5%	ND	ND	ND	ND	ND	ND
4	*Stenotrophomonas maltophilia* SBP-9	5-11	25–45	4% %	ND	ND	ND	ND	ND	ND
5	*Stenotrophomonas pavanii*	5-12	20–37	1.85%	ND	ND	ND	ND	ND	ND
6	*Stenotrophomonas rhizophila* e-p10	4-10	30	3%	ND	ND	ND	ND	ND	ND
7	*Stenotrophomonas maltophilia* DSM 21257	5-12	37	4.5%	ND	ND	ND	ND	ND	ND
8	*Stenotrophomonas chelatiphaga* LPM5	7-7.4	28	4%	ND	ND	ND	ND	ND	ND
9	*Stenotrophomonas indicatrix* WS40	6-9	10-37 °C	4%	ND	ND	ND	ND	ND	ND

Here ND: Not defined in the literature.

Supplementary Table S4: Genomic assembly statistics of *Stenotrophomonas maltophilia* BCM

Genome features	Values
**Genome size**	4519592 bp
**Contigs**	447
**CDS**	3949
**GC**	66.5
**rRNA**	7
**tRNA**	74
**tmRNA**	1
**N50**	139100bp

Supplementary Table S5: Average nucleotide identity (ANI) of *Stenotrophomonas maltophilia* BCM with other *Stenotrophomonas* species. Here the Bac1 to Bac12 represents the different *Stenotrophomonas* species represented as Bac1: *maltophilia* AU32848, Bac 2: *maltophilia* SM-1389, BAC 3: *pavanii* S18795, Bac 4: *rhizophila* Bl2-2, Bac 5: *rhizophila* GN_RF2.1, Bac 6: *rhizophila* JC1, Bac 7: rhizophila QL-P4, Bac 8: *pavanii* MHSD12, Bac 9: *pavanii* BWK1, Bac 10: *maltophilia* SMYN44, Bac 11: pavanii Y, Bac 12: *maltophilia* SMYN43, Bac 13: *acidaminiphila* BDBP 071, Bac 14: *bentonitica* VV6, Bac 15: *chelatiphaga* BIGb0227, Bac 16: *humi* DSM 18929, Bac 17: *nitritireducens* 2001.

	**BCM**	Bac 1	Bac 2	Bac 3	Bac 4	Bac 5	Bac 6	Bac 7	Bac 8	Bac 9	Bac 10	Bac 11	Bac 12	Bac 13	Bac 14	Bac 15	Bac 16	Bac 17
**BCM**	*	91.18 (82.94)	91.86 (83.44)	91.86 (83.44)	80.39 (60.83)	80.76 (61.75)	80.32 (61.55)	91.91 (85.55)	91.98 (85.44)	91.92 (85.4)	**99.57 (93.72)**	**97.56 (93.77)**	79.26 (48.18)	80.36 (61.41)	81.14 (59.09)	77.62 (48.99)	77.77 (50.08)	80.85 (59.35)
Bac1	91.19 (82.65)	*	91.96 (82.16)	90.78 (81.98)	80.43 (58.62)	80.67 (61.01)	80.41 (60.41)	90.89 (82.77)	90.91 (83.27)	90.82 (83.84)	**97.81 (94.76)**	**98.17 (95.06)**	79.24 (47.27)	80.41 (60.59)	81.13 (58.34)	77.74 (48.13)	77.83 (48.1)	80.86 (58.31)
Bac2	91.92 (83.4)	92.06 (82.27)	*	91.27 (81.54)	80.33 (60.29)	80.65 (61.15)	80.28 (60.89)	91.3 (82.79)	91.31 (83.93)	91.27 (83.95)	92.92 (88.01)	92.94 (88.01)	79.04 (47.97)	80.21 (61.6)	80.91 (59.24)	77.59 (48.27)	77.68 (48.98)	80.73 (58.86)
Bac3	91.72 (76.16)	90.64 (75)	91.02 (74.28)	*	80.54 (55.58)	80.8 (56.24)	80.46 (56.91)	**98.4 (83.26)**	**98.37 (83.96)**	**99.06 (87.95)**	91.81 (80.99)	91.86 (80.58)	79.39 (46.41)	80.58 (57.63)	81.29 (54.44)	77.49 (44.49)	77.95 (47.55)	81.12 (54.85)
Bac4	80.61 (59.46)	80.69 (57.87)	80.64 (58.93)	80.8 (59.71)	*	85.11 (63.35)	84.51 (63.52)	80.78 (60.16)	80.79 (60.58)	80.77 (60.46)	80.72 (61.33)	80.71 (61.23)	79.46 (46.17)	84.07 (64.04)	80.26 (59.38)	78.12 (47.38)	78.26 (49.22)	79.94 (55.42)
Bac5	80.98 (60.7)	81.08 (60.12)	81 (60.19)	81.24 (60.75)	85.1 (63.73)	*	85.65 (64.61)	81.16 (61.38)	81.11 (62.35)	81.13 (61.91)	81.14 (63.44)	81.13 (63.5)	80.02 (47.24)	85.83 (65.24)	80.9 (56.02)	78.58 (48.94)	78.72 (49.9)	80.29 (55.94)
Bac6	80.52 (61.67)	80.63 (60.74)	80.53 (60.66)	80.73 (62.43)	84.22 (64.89)	85.44 (65.41)	*	80.68 (62.44)	80.72 (62.68)	80.82 (63.08)	80.78 (63.91)	80.75 (63.41)	79.75 (50.42)	85.01 (67.21)	80.32 (57.25)	78.07 (50.13)	78.59 (53.03)	80.03 (57.84)
Bac7	91.96 (84.44)	90.95 (81.66)	91.2 (81.52)	**98.57 (89.99)**	80.6 (60.1)	80.97 (61.31)	80.46 (61.44)	*	**98.67 (92.38)**	**98.63 (92.59)**	92.1 (88.39)	92.07 (89.05)	79.29 (47.57)	80.38 (61.6)	81.24 (59.42)	77.56 (48.56)	77.85 (49.88)	80.97 (58.79)
Bac8	91.87 (84.01)	90.77 (82.05)	91.08 (82.41)	**98.44 (90.28)**	80.49 (60.33)	80.75 (62.29)	80.44 (61.48)	**98.62 (91.97)**	*	**98.55 (93.23)**	92.18 (89.03)	92.16 (89.25)	79.19 (47.49)	80.53 (60.84)	81.26 (58.97)	77.47 (48.1)	77.79 (49.28)	80.93 (58.51)
Bac9	91.79	90.7	91.02	**99.16**	80.62	80.88	80.61	**98.46**	**98.44**	*	92.13	91.82	79.48	80.72	81.22	77.62	77.95	81.14

	(80.31)	(79)	(78.79)	**(90.31)**	(57.82)	(58.94)	(59.02)	**(88.32)**	**(89.26)**		(86.48)	(85.17)	(45.71)	(59.23)	(57.24)	(46.27)	(47.82)	(57.23)
Bac10	**99.47 (94.68)**	94.61 (85.35)	91.76 (80.47)	91.16 (80.84)	80.29 (57.9)	80.57 (59.54)	80.35 (59.01)	91.23 (81.77)	91.36 (82.24)	91.35 (82.87)	*	**99.07 (95.05)**	79.2 (46.48)	80.36 (59.59)	81.08 (56.92)	77.6 (46.98)	77.82 (48.3)	80.92 (56.97)
Bac11	94.4 (86.45)	94.83 (86.52)	91.85 (81.64)	91.21 (81.73)	80.35 (59.01)	80.61 (60.51)	80.34 (59.9)	91.25 (83.67)	91.37 (83.87)	91.21 (83.76)	**99 (96.56)**	*	79.16 (46.83)	80.32 (60.06)	81.03 (58.03)	77.52 (47.87)	77.6 (48.58)	80.83 (57.6)
Bac12	79.63 (51.85)	79.7 (50.88)	79.5 (51.11)	79.92 (53.16)	79.67 (50.63)	80.15 (51.34)	79.95 (54.41)	79.78 (51.5)	79.77 (51.77)	79.88 (52.29)	79.96 (52.81)	79.89 (52.39)	*	79.52 (52.83)	78.9 (49.26)	81.81 (59.28)	82.07 (61.02)	78.6 (50.01)
Bac13	80.41 (60.9)	80.43 (60.88)	80.3 (60.78)	80.6 (62.83)	83.8 (64.83)	85.36 (65.73)	84.81 (67.39)	80.42 (62.04)	80.49 (62.12)	80.73 (63.6)	80.71 (64.35)	80.48 (63.02)	78.93 (49.74)	*	80.01 (59.72)	77.71 (49.94)	78.07 (51.63)	80.8 (61.63)
Bac14	81.1 (62.42)	81.19 (62.03)	81 (62.49)	81.37 (63.06)	80.22 (63.77)	80.76 (59.65)	80.24 (59.91)	81.31 (63.72)	81.29 (63.91)	81.32 (63.91)	81.23 (64.41)	81.23 (64.33)	78.7 (47.45)	80.12 (62.82)	*	77.32 (48.07)	77.38 (49.23)	83.96 (67.16)
Bac15	77.6 (52.17)	77.73 (52.1)	77.61 (51.99)	77.58 (52.43)	77.93 (51.87)	78.24 (53.17)	77.91 (53.69)	77.63 (52.45)	77.65 (52.74)	77.53 (52.79)	77.81 (53.53)	77.87 (53.36)	81.45 (58.4)	77.86 (53.01)	77.28 (49.25)	*	82.58 (68.66)	76.87 (49.77)
Bac16	77.72 (48.46)	77.77 (47.54)	77.67 (47.85)	77.96 (49.64)	78.03 (48.59)	78.48 (48.77)	78.61 (51.55)	77.8 (49.33)	77.82 (49.07)	77.97 (49.6)	78.24 (51.1)	77.89 (49.54)	81.91 (54.92)	78.12 (49.99)	77.28 (45.86)	82.54 (62.52)	*	77.17 (47.23)
Bac17	80.63 (59.33)	80.82 (58.73)	80.59 (59.18)	81.09 (60.26)	79.52 (57.25)	79.89 (57.12)	79.66 (58.01)	80.88 (59.79)	80.95 (59.9)	81.06 (60.91)	80.96 (62.09)	80.8 (61.4)	78.09 (47)	80.56 (62.38)	83.75 (63.84)	76.73 (47.03)	76.92 (48.37)	*

Supplementary Table S6: Tetra correlation among *Stenotrophomonas maltophilia* BCM and other *Stenotrophomonas* species by a wide distribution of Z-score

Organism	Z-Score
Stenotrophomona smaltophilia AU12-09	0.9996
Stenotrophomonas maltophilia Sm32COP	0.99954
Stenotrophomonas sepilia SM16975	0.99944
Stenotrophomonas maltophilia	0.99934
Stenotrophomonas maltophilia B1	0.99928
Stenotrophomonas sp. DDT-1	0.99904
Stenotrophomonas maltophilia D457	0.99901
Stenotrophomonas maltophilia A2	0.99897
Stenotrophomonas maltophilia ISMMS5	0.9989
Stenotrophomonas maltophilia EPM1	0.99884
Stenotrophomonas maltophilia UV74	0.99882
Stenotrophomonas maltophilia MF89	0.9988
Pseudomonas hibiscicola ATCC 19867	0.99878
Stenotrophomonas maltophilia As1	0.99878
Stenotrophomonas maltophilia K279a	0.99877
Stenotrophomonas maltophilia F2	0.99875
Stenotrophomonas maltophilia WJ66	0.99874
Stenotrophomonas maltophilia ISMMS4	0.99873
Stenotrophomonas maltophilia JV3	0.99872
Stenotrophomonas maltophilia	0.9986
*Stenotrophomonas maltophilia*OC194	0.99852
*Stenotrophomonas maltophilia*LMG 978	0.99851
*Stenotrophomonas maltophilia*Ab55555	0.9985
Stenotrophomonas geniculata ATCC 19374	0.99849
Stenotrophomonas maltophilia ISMMS2	0.99842
Stenotrophomonas maltophilia SM41	0.99839
Stenotrophomonas maltophilia NBRC 14161	0.99837
Stenotrophomonas geniculata N1	0.99836
Stenotrophomonas maltophilia MTCC 434	0.99836
Stenotrophomonas maltophilia CGMCC 1.1788	0.99835
Stenotrophomonas maltophilia ATCC 13637	0.99835
Stenotrophomonas sp. SKA14	0.9983
Stenotrophomonas pavanii DSM 25135	0.99828
Stenotrophomonas maltophilia SAM8	0.99823
*Stenotrophomonas maltophilia*13637	0.99822
Stenotrophomonas maltophilia R551-3	0.99815

Stenotrophomonas maltophilia NCTC10257	0.99813
Stenotrophomonas pavanii LMG 25348	0.99811
Stenotrophomonas maltophilia M30	0.99809
Stenotrophomonas maltophilia B5	0.99804
Stenotrophomonas maltophilia C11	0.99802
Stenotrophomonas maltophilia LMG 22072	0.99801
Stenotrophomonas maltophilia RA8	0.99758
Stenotrophomonas maltophilia ISMMS6	0.99721
Stenotrophomonas maltophilia Sm46PAILV	0.99703
Stenotrophomonas maltophilia SKK35	0.99654
Stenotrophomonas indicatrix WS40	0.9943
Stenotrophomonas maltophilia 5BA-I-2	0.99397
Stenotrophomonas sp. RIT309	0.99393
Stenotrophomonas lactitubi M15	0.99328
Stenotrophomonas maltophilia Sm41DVV	0.99147
Stenotrophomonas maltophilia B4	0.99135
Stenotrophomonas cyclobalanopsidis TPQG1-4	0.98493
Stenotrophomonas nematodicola CPCC 101271	0.97902
Stenotrophomonas rhizophila QL-P4	0.97624
Stenotrophomonas rhizophila DSM14405	0.97393
Stenotrophomonas maltophilia ZBG7B	0.97111
Stenotrophomonas sp. BIIR7	0.97069
Stenotrophomonas bentonitica DSM 103927	0.9705
Stenotrophomonas chelatiphaga DSM 21508	0.96999
Stenotrophomonas pennii Sa5BUN4	0.96969
Stenotrophomonas maltophilia JMNMN1	0.96745
Stenotrophomonas tumulicola JCM 30961	0.96492
Stenotrophomonas pictorum JCM 9942	0.96046
Stenotrophomonas sp. KCTC 12332 YM1	0.95856
Stenotrophomonas acidaminiphila DSM 13117	0.95586
Stenotrophomonas acidaminiphila JCM 13310	0.95453
*Stenotrophomonas acidaminiphila*ZAC14D2_NAIMI4_2	0.95452
Lysobacterruishenii CGMCC 1.10136	0.95441
Stenotrophomonas terrae DSM 18941	0.95352
Stenotrophomonas nitritireducens DSM 12575	0.95234
Stenotrophomonas sp. Leaf70	0.95196
Lysobacter daejeonensis GH1-9	0.9511
Stenotrophomonas panacihumi JCM 16536	0.95011
Stenotrophomonas humi DSM 18929	0.94757
Pseudomonas aeruginosa E15_London_28_01_14	0.94641
*Pseudoxanthomonas composti*GSS15	0.94515

Stenotrophomonas koreensis DSM 17805	0.94505
Pseudoxanthomonas mexicana DSM 17121	0.94442
Pseudoxanthomonas sp. Root630	0.94393
Pseudoxanthomonas sp. Root65	0.94228
Lysobacterprati SYSU H10001	0.93773
Pseudoxanthomonas japonensis DSM 17109	0.9377
Xanthomonas euroxanthea CPBF 424	0.93739
Pseudoxanthomonas spadix DSM 18855	0.93731
Pseudoxanthomonas spadix BD-a59	0.93603
Lysobacter selenitireducens 13A	0.93562
Lysobacter arenosi R7	0.93538
Pseudoxanthomonasgei KCTC 32298	0.93372
Lysobacterniastensis DSM 18481	0.93358
Lysobacterciconiae H21R20	0.93312
*Lysobacter*sp. A03	0.93263
Xanthomonas cannabis pv. cannabis	0.93261
Pseudoxanthomonas daejeonensis DSM 17801	0.93242
Xanthomonas cassavae CFBP 4642	0.93214
Stenotrophomonas ginsengisoli DSM 24757	0.932
Xanthomonas cassavae CFBP 4642 NCPPB 101	0.93125
Xanthomonas hyacinthi DSM 19077	0.93124
Lysobacter alkalisoli SJ-36	0.93124

Supplementary Table S7: Subsystem related protein features for the resistance of metal/metalloid, antibiotics and oxidative stress within the genome of *Stenotrophomonas maltophilia* BCM

Nature of resistance	Identified protein feature
Arsenic resistance	Arsenic resistance protein, ArsH, ACR3<br>Arsenic resistance operon repressor<br>Arsenate reductase
Cobalt-Zinc-Cadmium resistance	Cobalt-zinc-cadmium resistance protein CzcD
Hydroperoxide resistance	Organic hydroperoxide resistance protein
Resistance to antibiotics	Multidrug efflux system<br>small multidrug resistance family (SMR) protein

Supplementary Table S8: CAZymes encoding genes identified in *Stenotrophomonas maltophilia* BCM genome.

Sr no.	Enzyme family	Activities in family	Hits
1	Carbohydrate Esterase Family 1	Acetyl xylan esterase ; cinnamoyl esterase ; feruloyl esterase; carboxylesterase; S-formylglutathione hydrolase ; diacylglycerol O-acyltransferase ; trehalose 6-O-mycolyltransferase	7
2	Carbohydrate Esterase Family 16	Acetylesterase; active on various carbohydrate acetyl esters	1
3	GlycosylTransferase Family 1	UDP-glucuronosyltransferase ; zeatin O-β-xylosyltransferase ; 2-hydroxyacylsphingosine 1-β-galactosyltransferase; N-acylsphingosinegalactosyltransferase ; flavonol 3-O-glucosyltransferase; anthocyanidin 3-O-glucosyltransferase ; sinapate 1-glucosyltransferase ; indole-3-acetate β-glucosyltransferase ; flavonol L-rhamnosyltransferase ; sterol glucosyltransferase ; UDP-Glc: 4-hydroxybenzoate 4-O-β-glucosyltransferase; zeatin O-β-glucosyltransferase; limonoidglucosyltransferase ; UDP-GlcA: baicalein 7-O-β-glucuronosyltransferase; UDP-Glc: chalcone 4'-O-β-glucosyltransferase ; ecdysteroid UDP-glucosyltransferase; salicylic acid β-glucosyltransferase ; anthocyanin 3-O-galactosyltransferase ; anthocyanin 5-O-glucosyltransferase ; dTDP-β-2-deoxy-L-fucose: α-L-2-deoxyfucosyltransferase; UDP-β-L-rhamnose: α-L-rhamnosyltransferase ; zeaxanthinglucosyltransferase; UDP-Glc: flavone-6-C-glucosytransferase ; UDP-Glc: cinnamate β-glucosyltransferase ; UDP-Glc: hydroxycinnamic acid O-β-glucosyltransferase; UDP-Glc: cinnamoyl O-β-glucosyltransferase; UDP-Arap: flavone-C-arabinosytransferase ; [inverting] UDP-Glc: ginsenoside 3-O-glucosyltransferase; [inverting] UDP-Glc:	2

		3-O-glucosyl-protopanaxadiol-type ginsenoside 2"-O-glucosyltransferase; UDP-Glc: p-hydroxymandelonitrile-O-glucosyltransferase	
4	GlycosylTransferase Family 2	Cellulose synthase; chitin synthase; dolichyl-phosphate β-D-mannosyltransferase; dolichyl-phosphate β-glucosyltransferase); N-acetylglucosaminyltransferase; N-acetylgalactosaminyltransferase; hyaluronan synthase; chitin oligosaccharide synthase; β-1,3-glucan synthase; β-1,4-mannan synthase; β-mannosylphosphodecaprenol-mannooligosaccharide α-1,6-mannosyltransferase; UDP-Galf: rhamnopyranosyl-N-acetylglucosaminyl-PP-decaprenol β-1,4/1,5-galactofuranosyltransferase; UDP-Galf: galactofuranosyl-galactofuranosyl-rhamnosyl-N-acetylglucosaminyl-PP-decaprenol β-1,5/1,6-galactofuranosyltransferase; dTDP-L-Rha: N-acetylglucosaminyl-PP-decaprenol α-1,3-L-rhamnosyltransferase; alternating β-1,3/4-N-acetylmannan synthase (2.4.1.-); UDP-GlcA: N-acetylglucosaminyl-proteoglycan β-1,4-glucuronosyltransferase; [inverting] UDP-Glc: glycocin S-β-glucosyltransferase; [inverting] UDP-Glc: protein O-β-glucosyltransferase	11
5	Auxiliary Activity Family 10	AA10 (formerly CBM33) proteins are copper-dependent lytic polysaccharide monooxygenases (LPMOs); some proteins have been shown to act on chitin, others on cellulose; lytic cellulose monooxygenase (C1-hydroxylating; lytic cellulose monooxygenase (C4-dehydrogenating); lytic chitin monooxygenase; lytic xylanmonooxygenase / xylan oxidase (glycosidic bond-cleaving)	2
6	Glycoside Hydrolase Family 102	Peptidoglycan lytic transglycosylase	1

7	Carbohydrate Esterase Family 4	Acetyl xylan esterase; chitin deacetylase; chitooligosaccharidedeacetylase; peptidoglycan GlcNAcdeacetylase; peptidoglycan N-acetylmuramic acid deacetylase	4
8	Carbohydrate-Binding Module Family 32	Binding to galactose and lactose has been demonstrated for the module of Micromonosporaviridifacienssialidase. Binding to polygalacturonic acid has been shown for a Yersinia member. Binding to LacNAc (β-D-galactosyl-1,4-β-D-N-acetylglucosamine) has been shown for an N-acetylglucosaminidase from Clostridium perfingens	2
9	Glycoside Hydrolase Family 144	Endo-β-1,2-glucanase; β-1,2-glucooligosaccharide sophorohydrolase	1
10	Carbohydrate Esterase Family 16	Acetylesterase ; active on various carbohydrate acetyl esters	1
11	GlycosylTransferase Family 20	a,a-trehalose-phosphate synthase [UDP-forming] (EC 2.4.1.15); Glucosylglycerol-phosphate synthase (EC 2.4.1.213); trehalose-6-P phosphatase (EC 3.1.3.12); [retaining] GDP-valeniol: validamine 7-phosphate valeniolyltransferase	1
12	Glycoside Hydrolase Family 15	glucoamylase ; glucodextranase ; a,a-trehalase ; dextran dextrinase	1
13	GlycosylTransferase Family 51	murein polymerase	3
14	Glycoside Hydrolase Family 19	chitinase ; lysozyme ; [reducing end] chitinase	1
15	Glycoside Hydrolase Family 3	ß-glucosidase ; xylan 1,4-ß-xylosidase ; ß-glucosylceramidase ; ß-N-acetylhexosaminidase; a-L-arabinofuranosidase; glucan 1,4-ß-glucosidase ; isoprimeverose-producing oligoxyloglucan hydrolase ; coniferin ß-glucosidase ; exo-1,3-1,4-glucanase ; ß-N-acetylglucosaminidephosphorylases ; ß-1,2-glucosidase; ß-1,3-glucosidase ; xyloglucan-specific exo-ß-1,4-glucanase / exo-xyloglucanase; stevioside-ß-1,2-glucosidase ; lichenase / endo-ß-1,3-1,4-glucanase ; protodioscin 26-O-ß-D-glucosidase ; ß-glucuronidase ;	6

		avenacinase; tomatinase ß-1,2-glucosidase	
16	Carbohydrate Esterase Family 3	acetyl xylan esterase	1
17	Glycoside Hydrolase Family 23	lysozyme type G (EC 3.2.1.17); peptidoglycan lyase (EC 4.2.2.n1) also known in the literature as peptidoglycan lytic transglycosylase; chitinase	7
18	Glycoside Hydrolase Family 18	chitinase; lysozyme ; endo-ß-N-acetylglucosaminidase ; peptidoglycan hydrolase with endo-ß-N-acetylglucosaminidase specificity ; Nod factor hydrolase ; xylanase inhibitor; concanavalin B; narbonin; d?-N-acetylchitobiase / reducing-end exo-hexosaminidase ; chitobiosidase	2
19	Glycoside Hydrolase Family 31	a-glucosidase ; a-galactosidase; a-mannosidase; a-1,3-glucosidase ; sucrase-isomaltase ; a-xylosidase; a-glucanlyase ; isomaltosyltransferase ; oligosaccharide a-1,4-glucosyltransferase ; sulfoquinovosidase ; a-6-glucosyltransferase ; a-3-isomaltosyltransferase / cycloalternan-forming enzyme ; exo-acting protein-a-N-acetylgalactosaminidase	1
20	Carbohydrate Esterase Family 9	N-acetylglucosamine 6-phosphate deacetylase ; N-acetylgalactosamine 6-phosphate deacetylase	2
21	Carbohydrate Esterase Family 14	N-acetyl-1-D-myo-inosityl-2-amino-2-deoxy-a-D-glucopyranoside deacetylase (EC 3.5.1.89); diacetylchitobiosedeacetylase (EC 3.5.1.-); mycothiol S-conjugate amidase	1
22	Glycoside Hydrolase Family 5	endo-ß-1,4-glucanase / cellulase ; endo-ß-1,4-xylanase ; ß-glucosidase ; ß-mannosidase ; ß-glucosylceramidase ; glucan ß-1,3-glucosidase ; exo-ß-1,4-glucanase / cellodextrinase ; glucan endo-1,6-ß-glucosidase ; mannan endo-ß-1,4-mannosidase ; cellulose ß-1,4-cellobiosidase ; steryl ß-glucosidase ; endoglycoceramidase ; ß-primeverosidase ; xyloglucan-specific endo-ß-1,4-glucanase ; endo-ß-1,6-galactanase ; ß-1,3-mannanase; arabinoxylan-specific endo-ß-1,4-xylanase	

		; mannantransglycosylase ; lichenase / endo-ß-1,3-1,4-glucanase ; ß-glycosidase; endo-ß-1,3-glucanase / laminarinase ; ß-N-acetylhexosaminidase ; chitosanase ; ß-D-galactofuranosidase (EC 3.2.1.146); ß-galactosylceramidase ; ; ß-rutinosidase /a-L-rhamnose-(1,6)-ß-D-glucosidase; a-L-arabinofuranosidase ; glucomannan-specific endo-ß-1,4-glucanase; hesperidin 6-O-a-L-rhamnosyl-ß-glucosidase	
23	Glycoside Hydrolase Family 109	a-N-acetylgalactosaminidase ; ß-N-acetylhexosaminidase	3
24	Glycoside Hydrolase Family 103	peptidoglycan lytic transglycosylase	2

Supplementary Table S9: Hydrolases encoding genes identified in *Stenotrophomonas maltophilia* BCM genome.

Coding sequence Start position	Coding sequence Stop position	Strand	Function
		Protease	
48394	46490	-	Extracellular protease
85306	86430	+	Putative stomatin/prohibitin-family membrane protease subunit PA4582
44841	42898	-	Transglutaminase-like enzymes, putative cysteine proteases
55413	53128	-	ATP-dependent Clp protease ATP-binding subunit ClpA
56829	56503	-	ATP-dependent Clp protease adaptor protein ClpS
10443	11222	+	Zinc metalloprotease
132526	135375	+	FIG015547: peptidase, M16 family / FIG015287: Zinc protease
79357	81279	+	Signal peptide peptidase SppA (protease 4)
76447	74357	-	Catalase KatE-intracellular protease
106489	108021	+	Serine protease precursor MucD/AlgY associated with sigma factor RpoE
132552	132674	+	Serine protease
50816	52927	+	Protease II

56279	56830	+	ATP-dependent protease subunit HslV
56933	58306	+	ATP-dependent hsl protease ATP-binding subunit HslU
92903	95080	+	Tail-specific protease
78915	77482	-	Trypsin-like serine proteases, typically periplasmic, contain C-terminal PDZ domain
12794	13633	+	Intracellular protease
19268	20626	+	Intramembrane protease RasP/YluC, implicated in cell division based on FtsL cleavage
57431	58009	+	Uncharacterized protein, similar to the N-terminal domain of Lon protease
443	3	-	ATP-dependent Clp protease, ATP-binding subunit ClpC
3025	4497	+	Transglutaminase-like enzymes, putative cysteine proteases
		Lipase	
136521	137180	+	Phospholipase/carboxylesteras e family protein
170093	168240	-	Phospholipase/lecithinase/hem olysin
1	1062	+	FIG00613342: Bacterial patatin-like phospholipase domain containing protein

Supplementary Figure

Supplementary Figure SF1: Growth pattern analysis of *Stenotrophomonas maltophilia* BCM was observed after incubating the cultures for 48 hours in LB broth with constant shaking at 200 rpm. The experiment was carried out in triplicates, and growth was observed by taking absorbance at 600nm after 4 hours. Plotted values are the mean of triplicates along with the observed standard deviation.

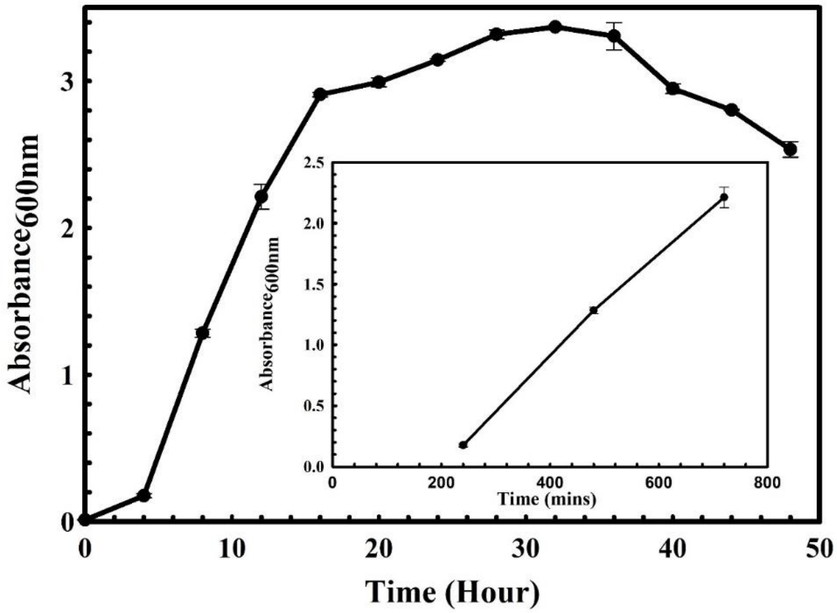

Supplement: Supplementary file 1 [file DataSheet_1.pdf]
